# Transcriptome alterations in spermatogonial stem cells exposed to bisphenol A

**DOI:** 10.1080/19768354.2022.2061592

**Published:** 2022-04-10

**Authors:** Jin Seop Ahn, Jong-Hyun Won, Do-Young Kim, Sang-Eun Jung, Bang-Jin Kim, Jun-Mo Kim, Buom-Yong Ryu

**Affiliations:** a Department of Animal Science & Technology, BET Research Institute, Chung-Ang University, Anseong-si, Republic of Korea; b Department of Cancer Biology, Perelman School of Medicine, University of Pennsylvania, Philadelphia, PA, USA

**Keywords:** Bisphenol A, RNA sequencing, spermatogonial stem cells, autophagy

## Abstract

Owing to their self-renewal and differentiation abilities, spermatogonial stem cells (SSCs) are essential for maintaining male fertility and species preservation through spermatogenesis. With an increase in exposure to plasticizers, the risk of endocrine-disrupting chemicals exerting mimetic effects on estrogen receptors, such as bisphenol A (BPA), has also increased. This has led to concerns regarding the preservation of male fertility. BPA impairs spermatogenesis and the maintenance of SSCs; however, the transcriptome differences caused by BPA in SSCs are poorly understood. Thus, this study aimed to investigate the transcriptome differences in SSCs exposed to BPA, using RNA sequencing (RNA-Seq) analysis. We found that cell proliferation and survival were suppressed by SSC exposure to BPA. Therefore, we investigated transcriptome differences through RNA-Seq, functional annotation, and gene set enrichment analysis. Our results showed repetitive and abundant terms related to two genes of lysosomal acidification and five genes of glycosaminoglycan degradation. Furthermore, we validated the transcriptome analyses by detecting mRNA and protein expression levels. The findings confirmed the discovery of differentially expressed genes (DEGs) and the mechanism of SSCs following exposure to BPA. Taken together, we expect that the identified DEGs and lysosomal mechanisms could provide new insights into the preservation of male fertility and related research.

## Introduction

Spermatogonial stem cells (SSCs) are important for male fertility and spermatogenesis (De Rooij 1998). SSCs are rarely present in the testes, comprising only 0.03% of germ cells in rodent testes (Tegelenbosch and de Rooij 1993). Despite the scarcity of the SSC population, it can maintain male fertility through self-renewal and differentiation. These abilities can simultaneously meet the requirements of maintaining stem cell pools and sperm production. SSCs are multiplied to germline cells through 9–11 mitotic divisions in spermatogenesis (De Rooij 2001). In a previous study, SSCs research had difficulties due to rare proportions of SSCs and morphological similarity compared to progenitor cells, which are abundant in the population (De Rooij and Russell 2000). To solve these issues, other studies have sought to determine a means for identifying phenotypic surface markers using in vitro culture conditions and confirmation through transplantation analysis (Brinster and Zimmermann 1994; Kubota et al. 2003, 2004). With the growing interest in SSCs, their importance has increased in male fertility preservation, transgenic animal production, genetic diseases, stem cell reprogramming and the potential of regenerative medicine (Guan et al. 2006; Ryu et al. 2007; Geens et al. 2008; De Rooij and Mizrak 2008; Lee Y et al. 2019b). Paternal genetic information is inherited only through spermatozoa, which are produced by SSCs. Therefore, it is valuable to understand the physiological properties of SSCs and factors from exoteric and intrinsic environments, such as toxic material and growth factors, that preserve their vitality.

Bisphenol A (2,2-bis(4-hydroxyphenyl) propane, BPA), an organic synthetic compound belonging to diphenylmethane derivatives and bisphenols, is globally used as a plasticizer to form polycarbonate plastics and epoxy resins for plastic material coatings. Manufacturing of BPA is one of the highest priorities for the plasticizer industry because it is cost-effective and convenient for production. The number of products synthesized using BPA as a plasticizer is increasing by 10% per year based on the principle of cost efficiency (Husain and Qayyum 2013). BPA is classified as an endocrine-disrupting chemical. It has an estrogen-mimicking effect and distinct mechanisms at estrogen receptor α (ERα) and ERβ (Gould et al. 1998; Pennie et al. 1998). Efforts to demonstrate the hazardous effects of BPA are progressing through investigation of its effects on imbalanced sex hormones and degradation of nuclear receptors and transgenerational effects on fertility in male germ cells (Tabb and Blumberg 2006; Karmakar et al. 2017). In addition, suppression of reproductive hormones is predominantly induced by exposing rodents and fish to BPA in vivo (Na et al. 2002; Jin et al. 2013). Furthermore, BPA exposure negatively affects sperm function, fertilization, and embryonic development (Rahman et al. 2015). It also negatively affects SSCs through apoptosis, autophagy, maintenance of the stem cell pool, and abnormal meiosis (Wang et al. 2010; Ali et al. 2014; Vrooman et al. 2015; Quan et al. 2017). These severe and hazardous problems associated with BPA are caused by its binding to the ERs located on SSCs (Saunders et al. 1998). Thus, further investigation of the negative effects of BPA is warranted. However, despite the importance of this avenue of research, transcriptome differences induced by the exposure of SSCs to BPA are poorly understood.

In this study, we investigated the effects of BPA on SSCs using high-throughput RNA sequencing (RNA-Seq) to identify differentially expressed genes (DEGs), and their application as biomarkers (Lee Y et al. 2019a). Through this study, we suggest a novel mechanism for the effect of BPA on SSCs, based on RNA-Seq and validation analyses.

## Material and methods

### Experimental animals

The use and management of experimental animals was approved by the Animal Care and Use Committee of Chung-Ang University (IACUC Number: 2019-00050) in accordance with the Guide for the Care and Use of Laboratory Animals of the National Institutes of Health.

### Isolation and culture of murine germline stem cells

C57BL/6-TgEGFP (C57GFP) mice were inbred from the same strain from the Jackson Laboratory (Sacramento, CA, USA). SSCs were isolated and cultured as previously described (Oatley and Brinster 2006), with minor modifications. To gather single cells from testes, chopped testes were incubated using a digestive solution consisting of a 2:1 ratio of 0.25% trypsin-ethylenediaminetetraacetic acid (EDTA) (Gibco, Waltham, MA, USA) diluted with 7 mg/mL DNase I (Roche, Basel, Switzerland) and Dulbecco's phosphate-buffered saline (DPBS) at 37°C for 5 min after pipetting several times. Following digestion, testicular cells were suspended and filtered through a 40 μm pore-size nylon mesh (BD Biosciences, San Jose, CA, USA). The collected pellet was washed with DPBS for sorting with magnetic-activated cell sorting (MACS) using CD90.2 microbeads (Miltenyi Biotech, Auburn, CA, USA). The culture plates had already been seeded with mitotically inactivated SIM mouse embryo-derived thioguanine- and ouabain-resistant (STO) feeder cells. Sorted cells were cultured in mouse serum-free medium (mSFM), containing a combination of growth factors (GF) consisting of 10 ng/mL glial cell line-derived neurotrophic factor (GDNF; R&D Systems, Minneapolis MN, USA), 75 ng/mL GDNF family receptor α 1 (GFRα1; R&D Systems), and 1 ng/mL basic fibroblast growth factor (bFGF; BD Biosciences), as previously described (Kubota et al. 2004).

### Treatment of SSCs with BPA

To evaluate the effect of BPA on SSCs, established SSCs were cultured with mSFM containing BPA (0.1% v/v) at previously described levels (0, 10, and 100 μM) (Karmakar et al. 2017)**.** BPA was diluted in dimethyl sulfoxide (DMSO) and the medium was replaced every 2 days. Proliferation and viability were evaluated using the trypan blue exclusion test. Three replicate experiments were performed for each treatment group.

### RNA-Seq

Total RNA concentration was calculated using Quant-IT RiboGreen (Invitrogen, Carlsbad, CA, USA). To assess the integrity of total RNA, samples were run on the TapeStation RNA screen tape (Agilent Technologies, Waldbronn, Germany). Only high-quality RNA preparations, with an RNA integrity number (RIN) greater than 7.0, were used for RNA library construction.

A library was independently prepared with 1 µg total RNA for each sample using the Illumina TruSeq Stranded Total RNA Sample Prep Kit (Illumina, Inc., San Diego, CA, USA). The rRNA in the total RNA was depleted using a Ribo-Zero kit. After rRNA depletion, the remaining RNA was purified, fragmented, and primed for cDNA synthesis. The cleaved RNA fragments were copied into first-strand cDNA using reverse transcriptase and random hexamers.

Then, second-strand cDNA synthesis was conducted using DNA polymerase I, RNase H, and dUTP. The synthesized cDNA fragments then underwent an end repair process via the addition of a single ‘A’ base, followed by ligation of the adapters. The products were then purified and enriched by polymerase chain reaction (PCR) to create the final cDNA library.

The libraries were quantified using quantitative reverse transcription-PCR (qRT-PCR) according to the qRT-PCR Quantification Protocol Guide (KAPA Library Quantification kits for Illumina Sequencing platforms) and qualified using the TapeStation D1000 Screen Tape (Agilent Technologies). Indexed libraries were then submitted to Illumina Hiseq X (Illumina, Inc.), and paired-end (PE, 2 × 151 bp) sequencing was performed by Macrogen Inc.

### DEG profiling

To select the quality filtering strategy of the RNA-Seq data, the raw PE sequencing data were examined using the FastQC (v0.11.7) program. The reads were trimmed with Trimmomatic (v0.38) (Bolger et al. 2014) to remove low-quality bases and adaptors. After this step, the quality of the reads was reevaluated using FastQC to confirm quality improvement. Subsequently, the high-quality reads were aligned to the mouse (mm10, Genome Reference Consortium Mouse Build 38) reference genome obtained from the Ensembl database (https://www.ensembl.org/) using Hisat2 (v2.1.0) (Kim et al. 2015). We used the default option during the alignment steps, using the Hisat2. SAMtools (v1.9), to convert the SAM file to a BAM file. Raw counts corresponding to genes in each library were calculated based on the exons of the genome annotation reference file Mus musculus GTF v100 (Ensembl) using featureCounts (v2.0.0) (Liao et al. 2014) from the Subread package. All DEG analyses for the raw counts were performed using the edgeR software package v3.24.3 of Bioconductor (Robinson et al. 2010). Genes with a raw quantification count of ≤10 were removed to reduce statistical bias in the DEG analyses. Since the count value of the quantified genes was an individually constructed library and sequenced for each dosage, normalized read counts were acquired using the trimmed mean of M-values (TMM) method by edgeR (v3.24.3) of the R package to improve raw quantitation counts. Multidimensional scaling (MDS) analysis was performed to compare the similarity between samples for the normalized value of each sample, and a plot was generated using the R package ggplot2 (v3.3.1). Normalized RNA-Seq values were examined for gene expression levels by comparing the control (0 μM) and BPA treatment groups (10 and 100 μM) using a negative binomial generalized linear model. The *P*-value was adjusted for multiple comparisons based on the Benjamini and Hochberg false discovery rate (FDR).

### Functional annotations and gene set enrichment analysis (GSEA)

The DEGs compared between each dosage were functionally annotated in the Kyoto Encyclopedia of Genes and Genomes (KEGG) and Gene Ontology (GO) using the Database for Annotation, Visualization, and Integrated Discovery (DAVID) bioinformatics resource 6.8 enrichment tool (Dennis et al. 2003; Jiang H et al.^2020^). In addition, enrichment analyses were filtered with the DIRECT option and applied with the following cutoffs: *P* < 0.1 and gene counts ≥ 2. KEGG pathway enrichment analysis was conducted, and the significant terms were represented by a fold enrichment level and a -log10 *P*-value. GO analyses were carried out in all three categories simultaneously, including biological processes, cellular components, and molecular functions. The REVIGO visualization tool was then used to create tree maps for the enriched GO terms.

To gain further insights into the biological roles of growth in an unbiased manner, we applied TMM values from the RNA-Seq data to GSEA (Subramanian et al. 2005). GSEA-identified genes that had comparatively enriched expression, compared to the control, were determined using a gene ranking method based on gene sets in the KEGG database. Furthermore, enrichment scores and statistically significant differences were determined using the GSEA v4.0.0 software. To estimate the statistical significance (nominal *P*-value) of the enrichment score (ES), we conducted an empirical 1,000 gene set permutation test procedure that preserves the complex correlation structure of the gene expression data. The ES is the maximum deviation from zero encountered in the random walk and use corresponding to a weighted Kolmogorov–Smirnov-like statistic. Then, the GSEA results were visualized via the bubble plot using R. The significant gene set is shown through an enrichment plot and heatmap of each gene (Kim et al. 2021).

### qRT-PCR

The RNA extraction procedure was performed by gelatin selection to exclude STO feeder cells from cultured cells. Total RNA was extracted using the Pure Link RNA mini kit (Invitrogen), and cDNA was synthesized using the Superscript IV reverse transcriptase (Invitrogen) according to the manufacturer’s instructions. Gene expression profiles were determined with qRT-PCR using the SYBR master mix (Applied Biosystems, Waltham, Massachusetts, USA) in ABI PRISM 7500 (Applied Biosystems). The relative expression level compared to the control group was evaluated using the 2^-ΔΔCT^ method. The conditions for gene amplification were as follows: holding stage (95°C for 10 min) followed by 45 cycles of 95°C for 15 s and 60°C for 1 min, and a final melting stage at 95°C for 15 s, 60°C for 1 min, 95°C for 30 s, and 60°C for 15 s. All primers used for the experiment are shown in Table S1.

### Western blot analysis

Proteins were extracted from SSCs cultured for one week with or without BPA using RIPA lysis buffer (89900, Thermo Scientific, Waltham, Massachusetts, USA) containing protease, phosphatase, and EDTA, and incubated for 30 min at 4°C. Lysates were centrifuged at 13,000 rpm for 15 min at 4°C and the supernatants were collected in fresh tubes. A bicinchoninic acid (BCA) assay (Thermo Scientific) was performed for protein quantification. Five micrograms of protein were electrophoresed on SDS-polyacrylamide gels and transferred onto a polyvinylidene difluoride (PVDF) membrane (Millipore, Billerica, MA, USA). The membrane was blocked with 5% skim milk in DPBS, containing 0.2% Tween-20 (PBS-T), at room temperature (21 ± 2°C) for 1 h. The membrane was then incubated with a 5% skim milk solution containing the primary antibody in a 1:5,000 dilution ratio, overnight at 4 °C. Mouse anti-α-tubulin was used as the loading control. After washing with PBS-T, the membrane was incubated with a 5% skim milk solution containing horseradish peroxidase (HRP)-conjugated secondary antibody, at room temperature (21 ± 2°C) for 1 h. Protein expression was determined with the ECL (Bio-Rad, Hercules, California, USA) method and measured in triplicates using the ImageJ software (US National Institutes of Health, Bethesda, MD, USA). Specific information on the antibodies is presented in Table S2.

### Statistical analysis

Statistical analyses were performed using GraphPad Prism 6 (GraphPad, La Jolla, CA, USA). Multiple comparisons were analyzed using the analysis of variance (ANOVA) method and Tukey’s honestly significant difference test. Tests were used to determine the differences among the treatment groups. The significance level was set at *P* < 0.05, and the values were expressed as mean ± standard error of the mean (SEM). All experiments were performed at least in triplicate.

## Results

### BPA adversely affects maintenance of SSCs

**Figure 1.**
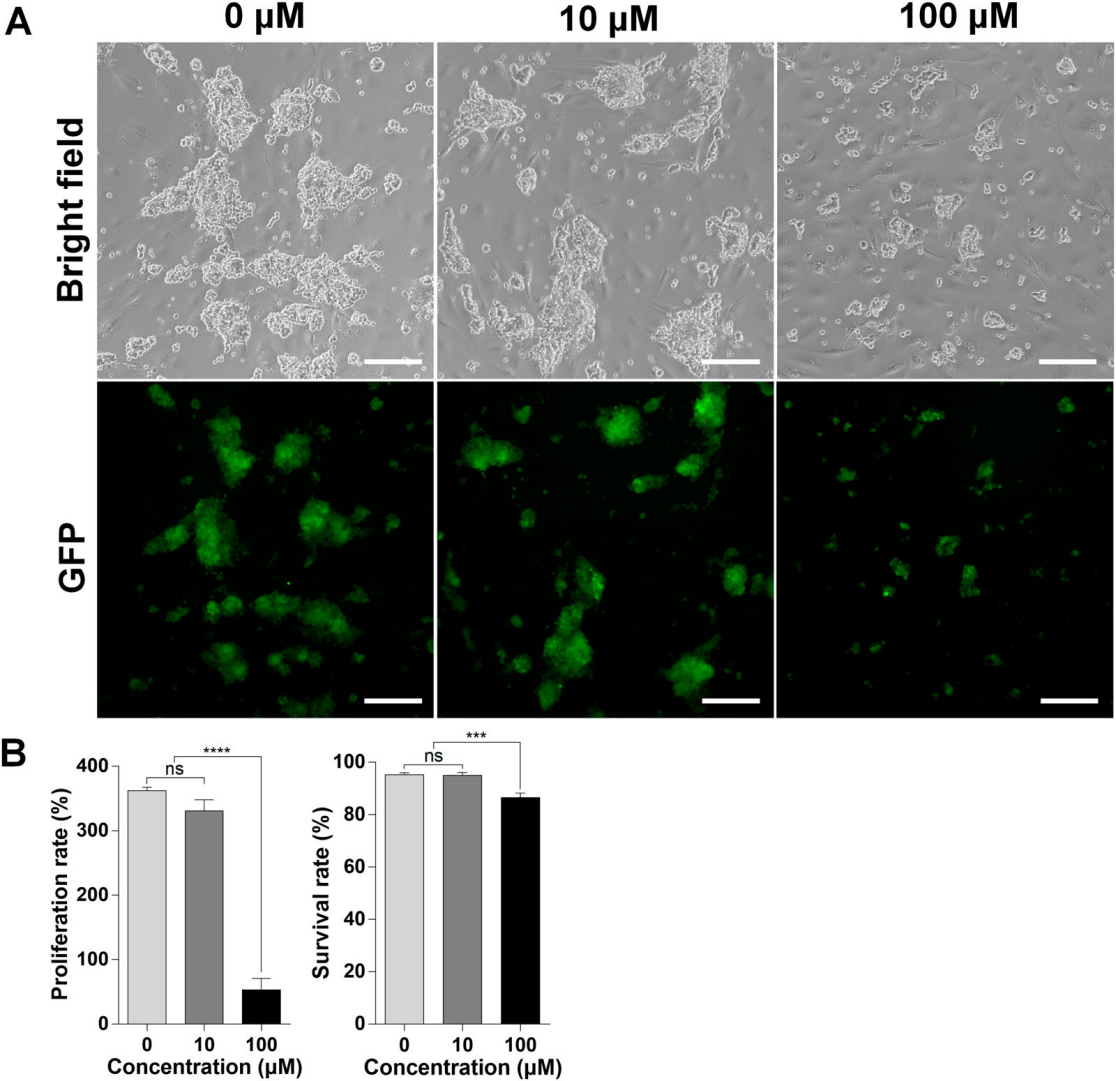
Effects of bisphenol A (BPA) on spermatogonial stem cells (SSCs) in vitro. (A) Effects of BPA on SSCs after culture for one week. BF, Bright field; GFP, green fluorescent protein. Scale bars = 200 μm. (B) The y-axis represents the proliferation rate and viability of BPA-treated murine SSCs. Cells were cultured with BPA for one week. Cell enrichment and survivability were calculated using a hemocytometer with the proper concentration of trypan blue dilution. Data were statistically analyzed by one-way analysis of variance (ANOVA). ns, no significant difference; ****P < 0.001 and **P < 0.01, respectively. Data are shown as mean ± standard error of the mean (SEM) (*n* = 3).

Isolated mouse SSCs were cultured with BPA for one week to evaluate the effect of BPA on SSCs. Both 0 µM and 10 µM BPA-treated SSCs appeared to have similar colony morphology; however, smaller colonies were observed after 100 μM BPA treatment than in the other groups (Figure 1(A)). These results indicated that SSCs and their proliferation ability were negatively affected by 100 μM BPA. To estimate the harmfulness of BPA treatment to SSCs, their proliferation rate and viability were measured using the trypan blue exclusion test (Figure 1(B)). The SSC proliferation rate was not significantly different at 10 µM BPA compared with that at 0 µM BPA. However, treatment with 100 μM BPA significantly decreased the proliferation rate compared to that in the other treatment groups (0 μM; 362.4 ± 8.6%, 10 μM; 330.6 ± 30.0%, 100 μM; 53.0 ± 30.7%). Cell viability was almost similar between the 0 μM and 10 μM BPA treatment groups; however, the SSCs treated with 100 μM BPA had significantly lower viability than those in the other treatment groups (0 μM; 95.2 ± 1.3%, 10 μM; 94.9 ± 2.1%, 100 μM; 86.5 ± 3.0%). These results demonstrate the adverse effects of BPA treatment on the proliferation and viability of SSCs.

### Overview of gene expression profiling for BPA treatment

**Figure 2.**
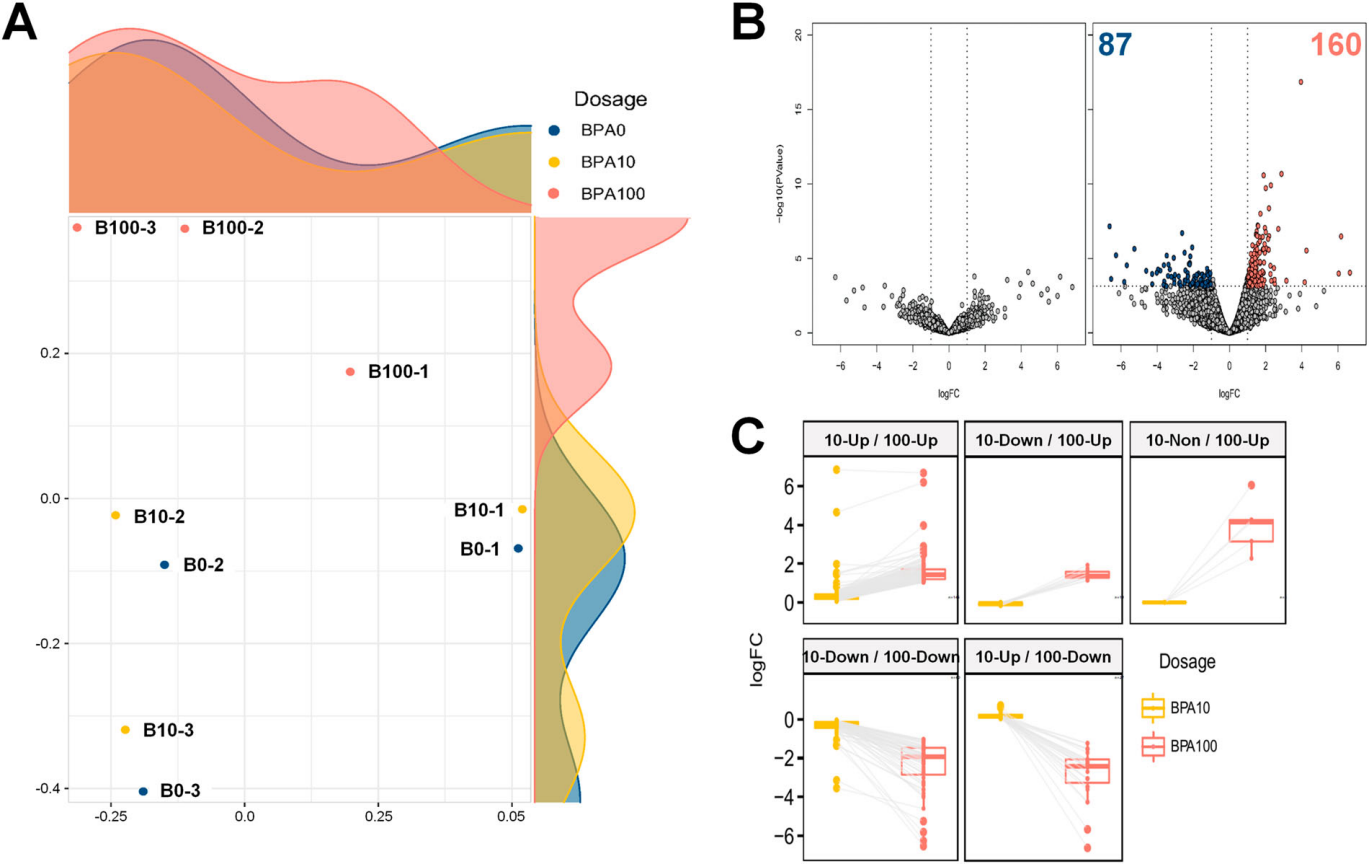
Differentially expressed genes (DEGs) in mouse germ cells, as assessed by RNA sequencing (RNA-Seq). (A) Multidimensional scaling (MDS) plot of the normalized trimmed mean of M-values (TMM) of RNA-Seq samples**.** MDS plot indicates cluster patterns of different samples. (B) Volcano plot indicates DEGs in spermatogonial stem cells (SSCs) at dosages of 10 and 100 μM bisphenol A (BPA). The color of each dot represents DEGs (navy and red) and nonsignificant DEGs (gray). (C) Box plot according to the direction of regulation of the expression of all DEGs.

RNA-Seq was performed to reveal the transcriptome changes induced by BPA treatment in SSCs. A total of 2.4 billion raw reads were produced from Illumina PE sequencing in nine samples of SSCs, with an average of 26.6 million reads per sample. The raw PE reads were trimmed by approximately 7.6% of the adaptor and low-quality reads through a quality control process. The reads that passed the trimming steps were aligned to the mouse reference genome, with an average mapping rate of 92.8% (ranging from 84.8–96.6%) (Table S3). According to the RNA-Seq data, the 100 μM-BPA treatment group had a more significant difference in the transcripts than that in the control and 10 μM-BPA treatment groups, and the 10 μM-BPA treatment group was clustered similarly to the control group (Figure 2(A)). DEGs were confirmed by comparing gene expression levels at each BPA dose (10 and 100 μM), relative to those at 0 μM (Figure 2(B)). DEGs were not found in the 10 μM-BPA treatment group, while a total of 247 DEGs were found in the 100 μM-BPA treatment group. Of these 247 DEGs, 87 were downregulated (Table S4) and 160 were upregulated (Table S5). When comparing DEGs from the 100 μM-BPA treatment group with the 10 μM-BPA treatment group, many of the genes had a comparable direction of regulation; although some genes were expressed in the opposite direction of regulation, they had a subtle expression (Figure 2(C)).

### Functional annotation analysis

**Figure 3.**
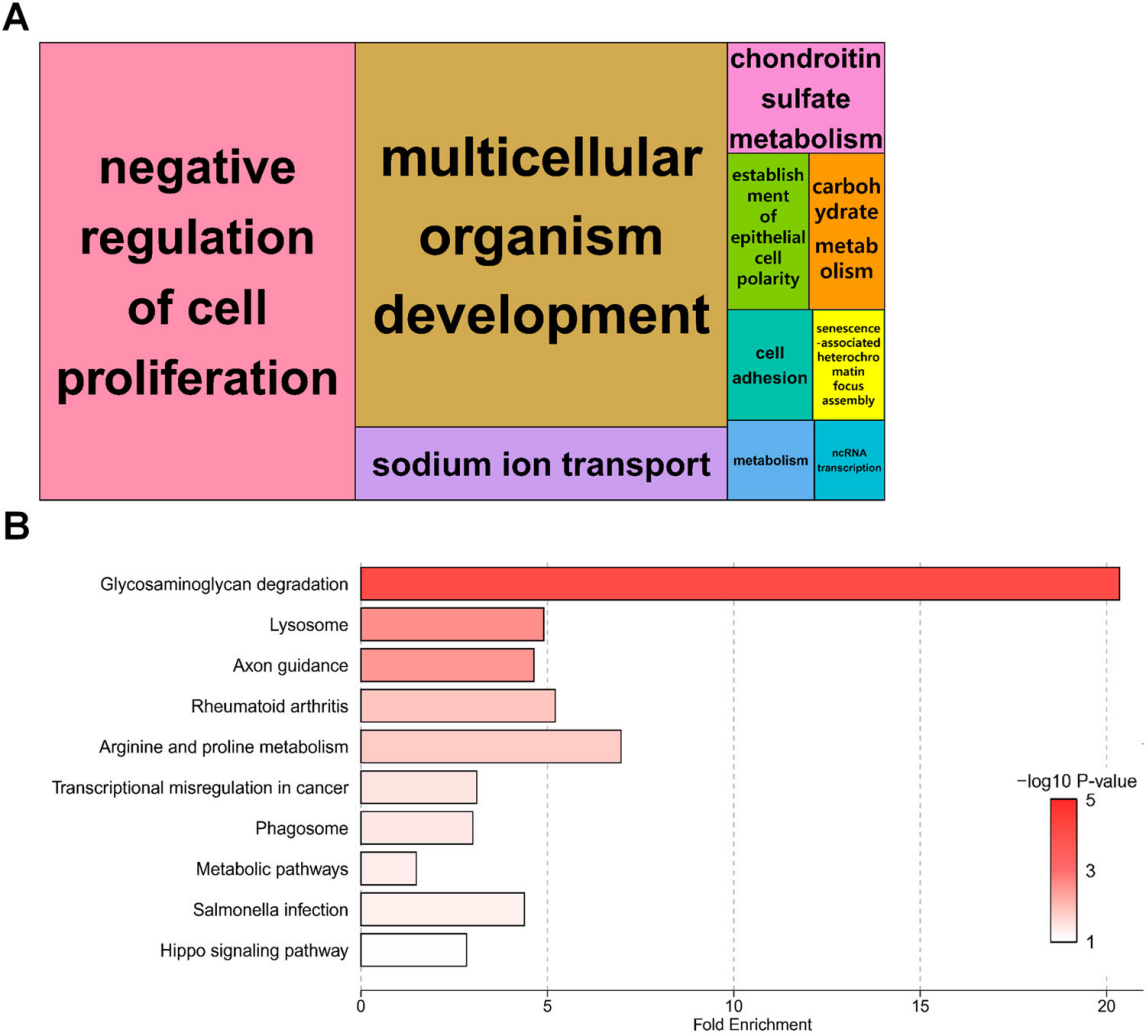
Enrichment analyses of biological significance for differentially expressed genes (DEGs) in spermatogonial stem cells (SSCs) exposed to 100 μM bisphenol A (BPA). (A) Tree maps for biological processes according to Gene Ontology (GO) analysis. Bold letters in the boxes represent DEGs enriched for representative terms. Larger boxes symbolize greater significance, and the box sizes are determined by the absolute value of the -log10 *P*-value. (B) Bar plot showing the fold enrichment and significance level for each Kyoto Encyclopedia of Genes and Genomes (KEGG) pathway term.

To gain a more comprehensive insight into the biological functions of BPA treatment, we performed GO and pathway analyses using DAVID. Details of the GO and KEGG pathway enrichment analyses are presented in Table S6. The biological process of GO analysis revealed significant enrichment of specific GO terms among the mRNAs differentially expressed in BPA-treated SCCs. DEGs were functionally enriched in ‘negative regulation of cell proliferation’, ‘multicellular organism development’, ‘sodium ion transport’, and ‘chondroitin sulfate metabolism’ (Figure 3(A)). A total of 52 DEGs were involved in the KEGG pathway enrichment analysis (44 upregulated DEGs, 8 downregulated DEGs), revealing that upregulated DEGs contributed to a greater extent. The KEGG pathway was the most abundant in the terms of ‘Glycosaminoglycan (GAG) degradation,’ followed by ‘Lysosome’. (Figure 3(B)).

### GSEA

**Figure 4.**
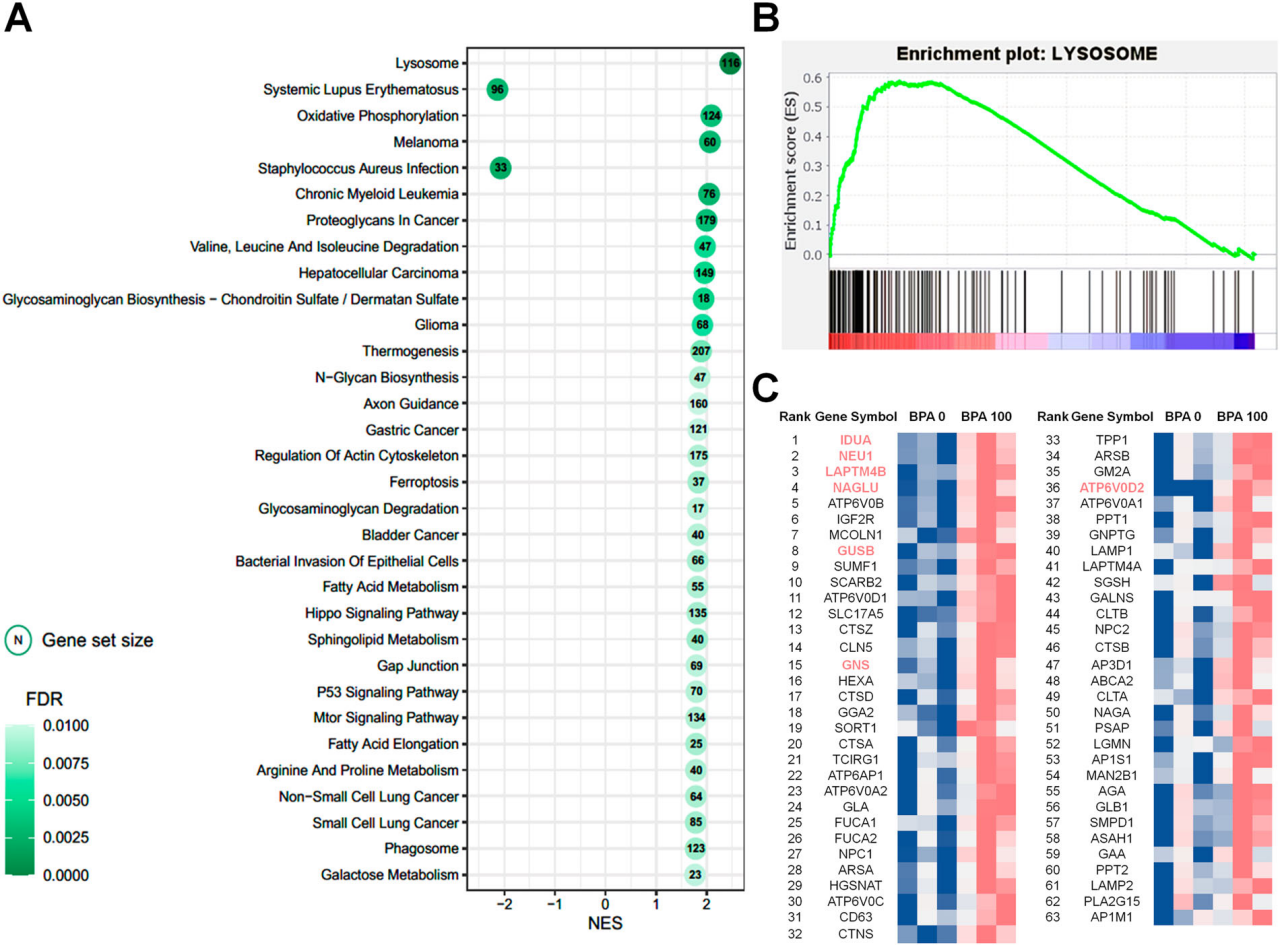
Gene set enrichment analysis (GSEA) based on the database of Kyoto Encyclopedia of Genes and Genomes (KEGG) pathways in spermatogonial stem cells (SCCs) treated with 100 μM bisphenol A (BPA). (A) Most significant functionally enriched KEGG pathway terms for SCCs treated with 100 μM BPA (*q*-value < 0.01). Dot size is proportional to the number of genes in the pathway. The color intensity of the dot (light to dark) indicates the level of significance. The *x*-axis indicates the normalized enrichment score (NES). (B) GSEA plot of the most significantly enriched pathways in SCCs treated with 100 μM BPA. (C) Subset of genes that have the most significant contributions to the lysosome gene sets. Bold and colored genes represent DEGs.

GSEA, based on the KEGG database, was performed using normalized TMM counts to identify differences in expression changes or expressed characteristics. The GSEA results revealed a similar dataset to that observed in the KEGG enrichment analyses of DEGs. Among the 32 significant pathways of GSEA, 30 terms had positive NES values and only two pathway terms (‘Systemic lupus erythematosus’ and ‘Staphylococcus aureus infection’) had negative NES values. Significant GSEA results (cutoff: q-value < 0.01) revealed various important pathway terms, such as ‘Melanoma’, ‘Chronic myeloid leukemia’, ‘Hepatocellular carcinoma’, ‘Glioma’, ‘Gastric cancer’, and ‘Bladder cancer’, which are directly or indirectly related to cancer. In addition, terms related to autophagy, such as ‘Lysosome’ and ‘Phagosome’, were identified; among these, ‘Lysosome’ showed the highest NES and the most significant value (Figure 4(A,B)). Of the total genes expressed in the 100 μM-BPA treatment group gene set, only 116 contributed to the lysosome pathway. Among these genes, the expression levels of 63 key enriched genes, which make significant contributions, were visualized by generating a heatmap (Figure 4(C)). Among these 63 genes, seven DEGs (IDUA; Alpha-L-Iduronidase, NEU1; Neuraminidase 1, LAPTM4B; Lysosome-associated Protein Transmembrane 4 Beta, NAGLU; N-Acetyl-Alpha-Glucosaminidase, GUSB; Glucuronidase Beta, GNS; Glucosamine (N-Acetyl)-6-Sulfatase, and ATP6V0D2; adenosine triphosphatase H+ transporting V0 subunit D2) were identified. Most of these genes were found to have the highest enrichment contribution to lysosomes. Furthermore, it was confirmed that the *ATP6V0D2* gene, which has the highest fold change level among DEGs involved in the corresponding pathway, does not show a high enrichment order, but contains various genes that produce the identical subunit protein V-type proton ATPase produced by *ATP6V0D2*. Pathview was used to map and display a set of 100 μM BPA-associated DEGs on a lysosome pathway graph (Figure S1). Among the total genes involved in the lysosome pathway (122), several genes (116) expressed through the 100 μM BPA treatment contributed to transcriptional changes. In addition, in the KEGG pathway, ATPeV (ATP6V0D1, ATP6V0A1, ATP6V0C, ATP6V0A2, TCIRG1, ATP6AP1, ATP6V0B, and ATP6V0D2) showed high levels of gene expression following BPA treatment.

### RNA-Seq validation using qRT-PCR

**Figure 5.**
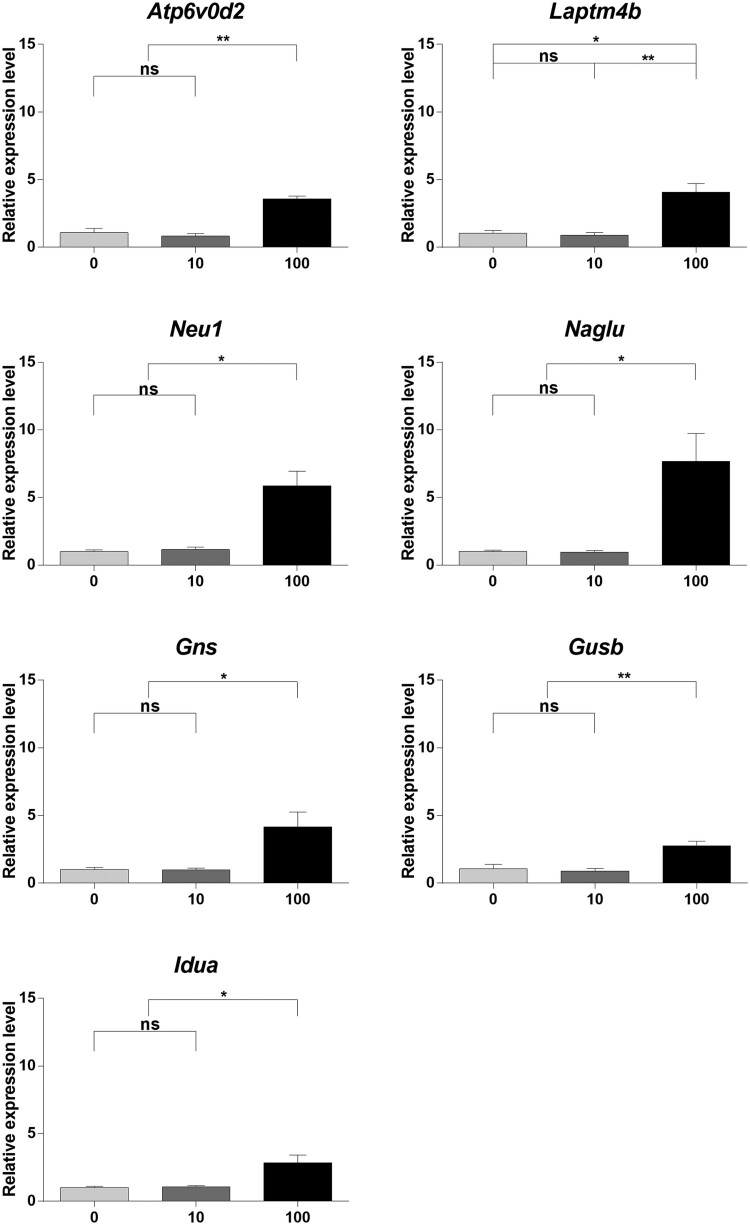
Gene expression profiling using quantitative real-time polymerase chain reaction (qRT-PCR). (A) Relative gene expression was measured using qRT-PCR. Samples were collected after culturing spermatogonial stem cells (SSCs) with bisphenol A (BPA) for one week. Differences in gene expression were normalized to the 0 μM-BPA treatment group as fold change. The x-axis shows the BPA concentration. Data were statistically analyzed by one-way analysis of variance (ANOVA). ns, no significant difference; *P < 0.05 and **P < 0.01. Data are shown as mean ± standard error of the mean (SEM) (*n *= 3).

After RNA-Seq analysis, qRT-PCR was performed on seven genes (*Atp6v0d2, Laptm4b, Neu1, Idua, Naglu, Gusb,* and *Gns*) to validate the equivalence between RNA-Seq data and transcriptome differences in the BPA-treated mouse SSCs. The results of qRT-PCR showed that the selected genes were no significant differences between the 0 μM and 10 μM BPA-treated groups; however, the 100 μM BPA-treatment showed significant differences in all selected genes compared to the other concentrations of BPA (Figure 5). These results revealed that 100 μM BPA treatment caused transcriptome differences in the mouse SSCs. According to RNA-Seq analysis, all selected genes were upregulated by treatment with 100 μM BPA. The equivalence of the assessed RNA-Seq data and transcriptome differences indicates that RNA-Seq could reliably represent transcriptome differences.

### RNA-Seq validation using western blot analysis

**Figure 6.**
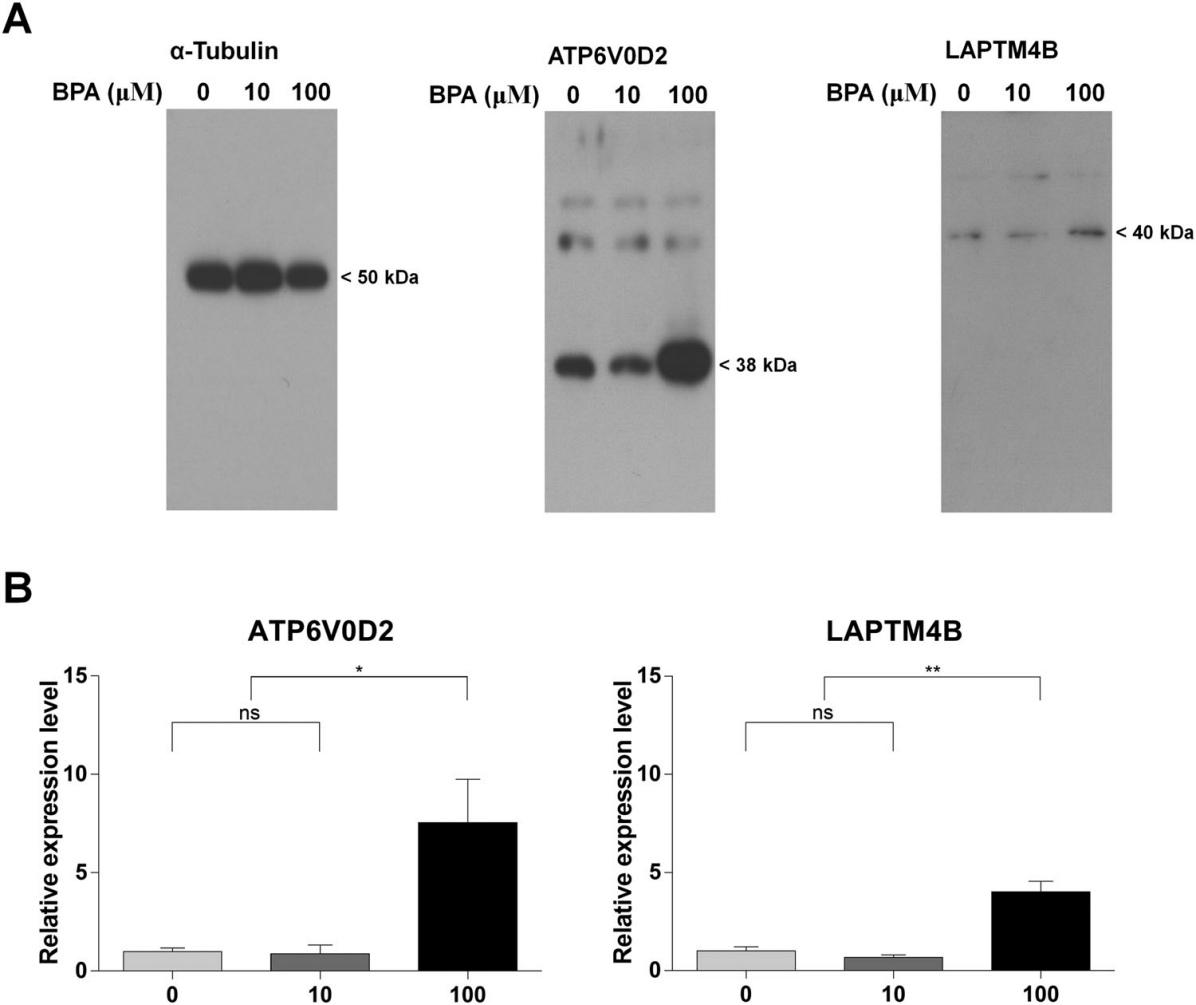
Different protein expression patterns induced by bisphenol A (BPA) treatment. (A) Western blot analysis of developed and fixed protein patterns. The text at the top shows the protein name and BPA concentration. Protein sizes are shown on the right. (B) The ratio of protein expression was calculated between the control and BPA treatment groups according to the relative expression level. Each difference in protein expression was normalized to that of the 0 μM-BPA treatment group as fold change. The x-axis represents the BPA concentration. Data were statistically analyzed by one-way analysis of variance (ANOVA). ns, no significant difference; *P < 0.05 and **P < 0.01. Data are shown as mean ± standard error of the mean (SEM) (*n* = 3).

We confirmed the protein expression of ATP6V0D2 and LAPTM4B, which were simultaneously detected in the previous results with the term ‘Lysosome’. The sizes of ATP6V0D2 and LAPTM4B were 38 kDa and 40 kDa, respectively (Figure 6(A)). Protein expression levels were quantified and normalized using a quantified level of α-tubulin. The relative expression levels of the two genes were not significantly different between the 10 μM-BPA and the 0 μM-BPA groups. In contrast, the genes were significantly expressed in the 100 µM-BPA group compared to that in the 0 μM- and 10 μM-BPA groups (Figure 6(B)). These results verified that the ATP6V0D2 and LAPTM4B proteins were upregulated in the mouse SSCs after treatment with 100 μM BPA.

## Discussion

In this study, we demonstrated the effects of BPA on mouse SSCs at the transcriptome level using RNA-Seq analysis. Hormonal mimic effects of BPA can occur in spermatogonia and spermatocytes because of the estrogenic characteristics of BPA and the expression of ERβ in spermatogonia and spermatocytes (Saunders et al. 1998). Karmakar et al. (2017) also found that BPA had negative effects on mouse SSCs, leading to low proliferation and cell viability at high doses. The malignant effects induced by BPA have been actively investigated because of the risk these effects pose to the reproductive system, apoptosis-induced abnormality, and hormone disorders (Tabb and Blumberg 2006; Wang et al. 2012; Gurmeet et al. 2014). These studies suggest that the endocrine-disrupting effects of BPA could cause critical problems in male fertility. Therefore, such research is important for understanding and maintaining male reproductive ability, which is essential for species preservation.

In our previous study, BPA could alter the proliferation rates, apoptosis rates, and stemness of SSCs. Thus, the treatment concentrations of BPA were determined to be 10 and 100 μM, as previously described (Karmakar et al. 2017). At 100 μM BPA, SSCs showed morphological differences with small colonies compared to that at 0 and 10 μM BPA. Furthermore, we could recognize the following terms related to SSC maintenance through GO term analysis: ‘Negative regulation of cell proliferation’ and ‘Cell adhesion’. To clarify this observation, the effects of BPA on the proliferation rate and viability of SSCs were determined. The measured proliferation and survivability could represent SSC suppression. These findings reveal that the maintenance of SSCs is inhibited by a high concentration of BPA.

Upon DEG analysis, we observed no DEGs in the SCCs treated with 10 μM BPA, whereas 247 DEGs were observed in SSCs treated with 100 μM BPA. In addition, different DEG regulation patterns were observed between SSCs treated with 10 and 100 μM BPA. For this reason, we examined whether transcriptomes were significantly affected by 100 μM BPA treatment and not by 10 μM BPA treatment.

Using GO term analysis, terms including ‘sodium ion transport’, ‘chondroitin sulfate metabolism’, and ‘carbohydrate metabolism’ were found to be related to lysosomal functions (Rome and Hill 1986; Beyenbach and Wieczorek 2006; Wang et al. 2012; Xiong and Zhu 2016). Our results showed that diverse functions were simultaneously promoted by BPA.

To obtain more accurate biometric information, we performed KEGG pathway enrichment analysis. Enriched KEGG pathways were strongly and significantly involved in ‘GAG degradation’ and ‘Lysosome’ pathways. The terms ‘Arginine and proline metabolism’ and ‘Phagosome’ pathway enrichment were also observed, which are related to lysosomal maturation (Yates et al. 2005; Pauwels et al. 2017) and functions (Ernst et al. 1995; Abu-Remaileh et al. 2017). The other terms were distinct from lysosomal activity. Among them, the ‘Hippo signaling’ pathway is known to regulate organ size and stem cell proliferation (Ramos and Camargo 2012). Therefore, enriched KEGG pathways abundantly represented lysosome-related terms. By combining these functional annotations, we found abundant and repetitive lysosome-associated terms and pathways.

GSEA was performed to identify whether it is statistically significant based on the KEGG database and normalized TMM counts. The results of GSEA showed that the highest and most significant NES value was detected for the term ‘Lysosome’, while GAG-related terms had relatively low values. Using GO terms, KEGG pathway enrichment analysis, and GSEA, we noticed that lysosome-related terms were represented repeatedly in various analyses. Lysosomes can adapt to different environments via biogenesis and functions through global transcriptional regulation (Settembre et al. 2013). Furthermore, lysosomes can regulate cell proliferation and tissue regeneration (Shin and Zoncu 2020) which are promoted by lysosomal acidification via ion transportation (Yambire et al. 2019). Therefore, we investigated lysosomal transcriptomes to reveal the relationship between lysosomes and the adverse effects of BPA exposure on murine SSCs.

Using our previous analyses, we found that seven DEGs were included in the ‘Lysosome’ term. The selected DEGs were represented in the lysosome membrane and lumen, which can be grouped as lysosome acidification or GAG degradation. ATP60VD2 and LAPTM4B are involved in lysosomal acidification. ATP6V0D2 is a membrane-bound subunit of vacuolar ATP synthase (V-ATPase) that transports H+ ions into lysosomes and affects lysosome/autophagosome membrane fusion (Xia et al. 2019). Transportation of H+ ions into lysosomes is an important process for lysosomal acidification, as lysosomal activity is determined by luminal pH because of its unique role (Colacurcio and Nixon 2016). Moreover, LAPTM4B is mainly located in the lysosomal membrane. It has the ability to activate V-ATPase and mTORC1 (mammalian target of rapamycin complex 1) at the lysosome through leucine transport (Milkereit et al. 2015). In addition, lysosomal acidification, which is induced by V-ATPase activation, may affect SSC proliferation and differentiation through mTORC1 (Puertollano 2014; Serra et al. 2017). These two DEGs were validated using qRT-PCR and western blotting. Through these analyses, we found that these DEGs had similar regulation patterns. The other identified DEGs were involved in GAG degradation and comprised *Idua*, *Neu1*, *Naglu*, *Gusb*, and *Gns*. These DEGs were strongly related to heparan sulfate degradation. Heparan sulfate has been shown to promote GDNF signaling (Barnett et al. 2002; Langsdorf et al. 2011) and FGF signaling (Schlessinger et al. 2000) in vivo, which is critical for SSC maintenance (Takashima et al. 2015). In contrast, upregulation of *Neu1* is a result of GAG degradation (Kreutz et al. 2013). Taken together with the data from previous studies, these findings demonstrate that upregulation of lysosomal acidification is a negative signal for SSC maintenance.

**Figure 7.**
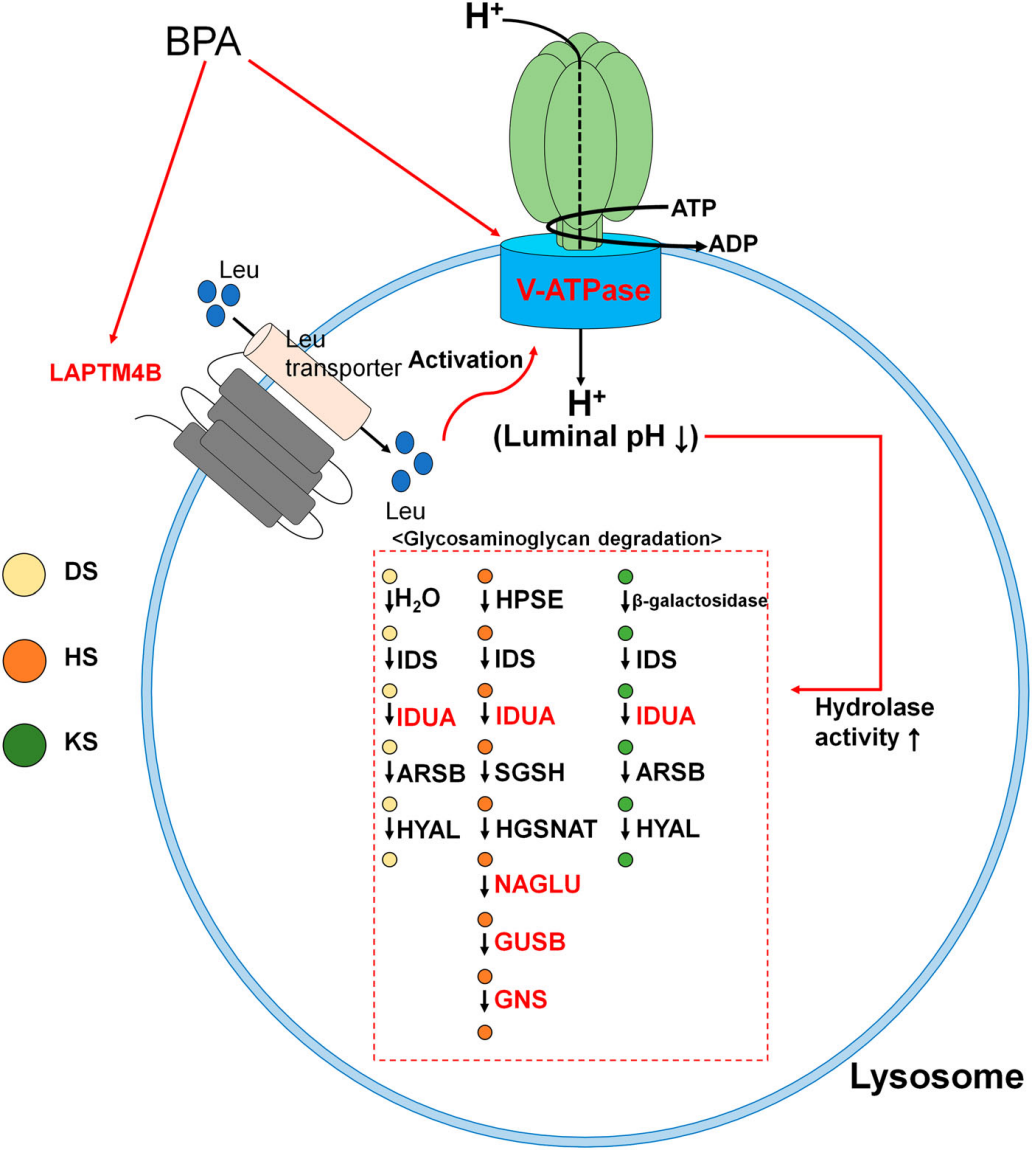
Overall process resulting from bisphenol A (BPA) exposure. Figure represents the cascade of lysosomal processes caused by exposure to BPA. DS; dermatan sulfate, HS; heparan sulfate, KS; keratan sulfate. Red text represents recognized differentially expressed genes (DEGs).

Thus, we conducted a western blotting analysis on ATP6V0D2 and LAPTM4B to confirm whether transcriptome differences influenced their protein expression patterns. This was done because lysosomal acidification and maturation are essential processes for GAG degradation. As shown in Figure 6, both ATP6V0D2 and LAPTM4B showed significant differences when SSCs were treated with 100 μM BPA compared with the control, as shown in RNA-Seq. Thus, we can infer that lysosomal changes occur as a result of BPA treatment. We suggest that GAG degradation is upregulated by the cascading processes of lysosomal acidification. The entire process is organized in Figure 7 based on these results. Therefore, we suggest that the ‘Cell proliferation rate’ and ‘Negative regulation of cell proliferation’ terms might be induced by heparan sulfate degradation processes via the upregulation of *Idua*, *Neu1*, *Naglu*, and *Gusb*. Moreover, this cascade may be activated by the upregulation of lysosomal acidification through upregulation of ATP6V0D2 and LAPTM4B.

In summary, we discovered transcriptome differences in SSCs exposed to BPA using RNA-Seq. BPA induced a reduction in cell proliferation through GAG degradation and lysosomal acidification. Most importantly, our findings suggest that BPA affects the lysosomes of SSCs by upregulating the transcriptome and protein levels of ATP6V0D2 and LAPTM4B. This result is beneficial for elucidating the lysosomal mechanism and membrane markers to confirm lysosomal changes induced by BPA. Furthermore, we expect that knowledge of this mechanism could contribute to the field of reproductive medicine and male infertility.

## Supplementary Material

Supplemental Material
