# Supplementary material for: Transcriptome alterations in spermatogonial stem cells exposed to bisphenol A
Source: Anim Cells Syst (Seoul). 2022 Apr 10;26(2):70–83. doi: 10.1080/19768354.2022.2061592 (PMC9037227; doi:10.1080/19768354.2022.2061592)
Supplement: Supplemental Material [file TACS_A_2061592_SM1025.docx]

**Transcriptome alterations in spermatogonial stem cells exposed to bisphenol A**

Supplemental information

**Supplementary figure 1.** Pathview analysis of lysosomes.

**Supplementary table 1.** List of primer sequences for qRT-PCR

**Supplementary table 2.** List of antibodies for western blotting

**Supplementary table 3.** Summary of RNA sequencing data

**Supplementary table 4.** Differentially expressed genes (DEGs) downregulated between mouse SSCs treated with 100 μM BPA and 0 μM BPA

**Supplementary table 5.** Differentially expressed genes (DEGs) upregulated between mouse SSCs treated with 100 μM BPA and 0 μM dosage.

**Supplementary table 6.** GO and KEGG pathway enrichment analyses of DEGs in SSCs treated with 100 μM BPA

**
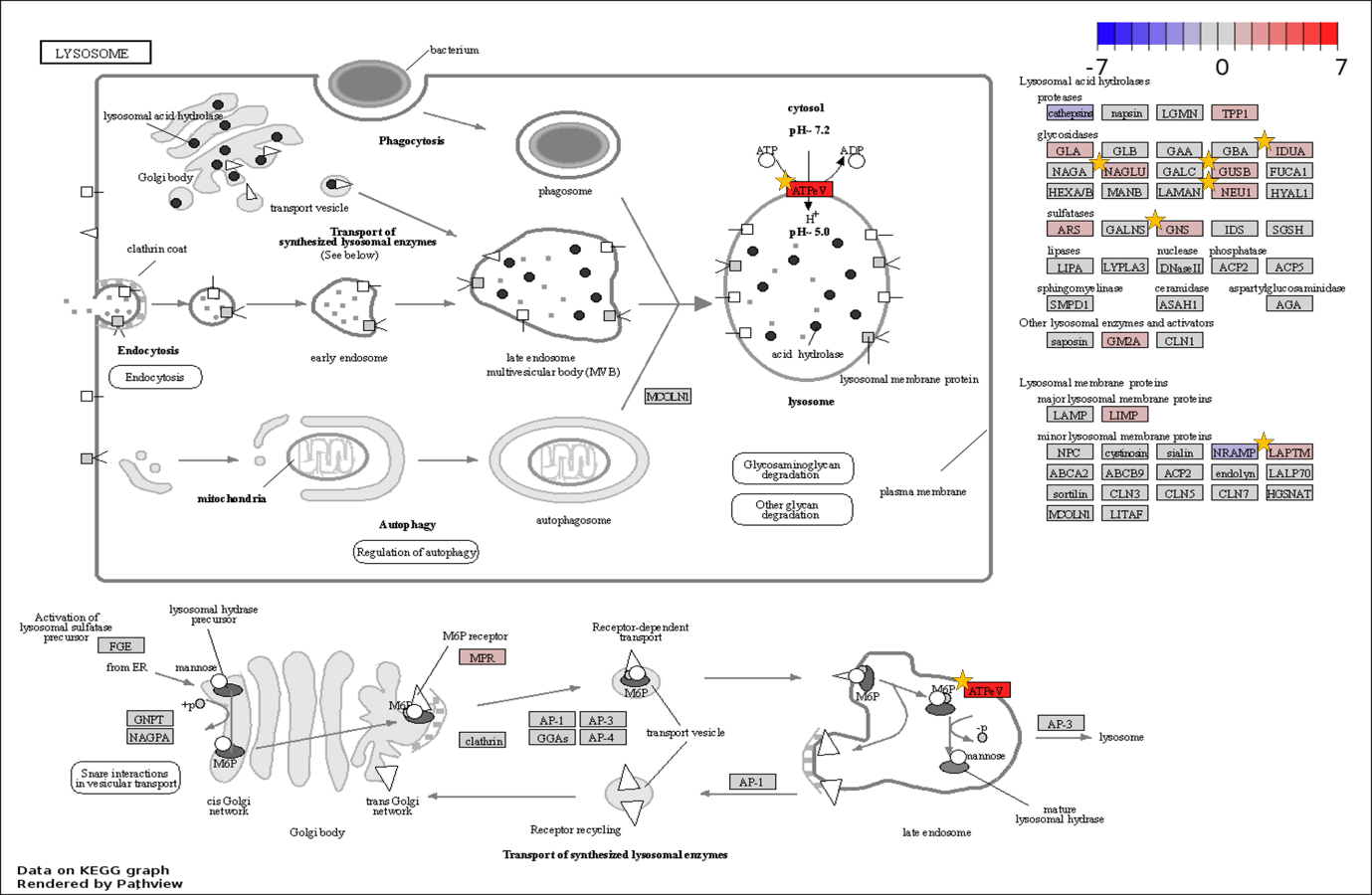
**

**Supplementary figure 1.** Pathview analysis of lysosomes. The color of the gene indicates the expression level. The stars marked on the genes symbolize differentially expressed genes (DEGs).

**Supplementary table 1.** List of primer sequences for qRT-PCR

| **Gene** | **Sequences (5' → 3')** | **Accession number** |
| --- | --- | --- |
| *Gapdh* | F: TGAAGGTCGGTGTGAACGG | NM_008084.3 |
|  | R: CGTGAGTGGAGTCATACTGGAA |  |
| *Atp6v0d2* | F: GGCTCTATCCAGAGGGGTTG | NM_175406.3 |
|  | R: GCGTCAAACAAAGGCTTGTA |  |
| *Laptm4b* | F: TCCGTGAATCCTACCTGTTTG | NM_033521.3 |
|  | R: GTTCCTGCCGTTGATGTACC |  |
| *Idua* | F: GACCTTAGTTGGGACCAGCA | NM_008325.4 |
|  | R: GGTGACTTCCTGGCTGTGAT |  |
| *Gusb* | F: CTTTATTGGCTGGGTGTGGT | NM_010368.2 |
|  | R: AATGGGCACTGTTGATCCTC |  |
| *Neu1* | F: GGAAGCAGATCGGCTCTGTA | NM_010893.3 |
|  | R: CCTCATCGGATGCAGATTTT |  |
| *Naglu* | F: TGACTTCTGTGGCTGTCAGG | NM_013792.2 |
|  | R: GCGTGCACACATTCTGGTAA |  |
| *Gns* | F: AAGAAAACCAAGGCCCTCAT | NM_029364.4 |
|  | R: TGGGGGTACTTTCCAGTCAG |  |

**Supplementary table 2.** List of antibodies for western blotting

| **Primary antibody** | **Dilution rate** | **Company** | **Catalog number** |
| --- | --- | --- | --- |
| Mouse monoclonal α-Tubulin | 5000 :1 | Abcam | ab7291 |
| Rabbit polyclonal ATP6V0D2 | 5000 :1 | Thermo Fisher Scientific | PA5-44359 |
| Rabbit polyclonal LATPM4B | 5000 :1 | Thermo Fisher Scientific | PA5-43047 |
| **Secondary antibody** | **Dilution rate** | **Company** | **Catalog number** |
| Anti-mouse HRP-linked antibody | 2000:1 | Cell Signaling Technology. | 7076s |
| Anti-rabbit HRP-linked antibody | 2000:1 | Cell Signaling Technology. | 7074s |

**Supplementary table 3.** Summary of RNA sequencing data

| **Group** | **Sample** | **Raw reads** | | **Trimmed reads** | | **Mapping** |
| --- | --- | --- | --- | --- | --- | --- |
|  |  | **Total sequences** | **GC%** | **Total sequences** | **GC%** | **Total (%)** |
| 0 μM BPA | B0-1 | 25,544,665 | 46 | 24,247,858 | 45 | 23,399,183 (96.5) |
|  | B0-2 | 27,309,544 | 45 | 25,331,436 | 45 | 21,701,441 (85.7) |
|  | B0-3 | 25,254,576 | 45 | 23,132,898 | 44 | 20,983,852 (90.7) |
| 10 μM BPA | B10-1 | 24,504,185 | 47 | 22,612,108 | 46 | 21,841,035 (96.6) |
|  | B10-2 | 29,053,464 | 44 | 26,867,290 | 44 | 22,772,715 (84.8) |
|  | B10-3 | 25,606,157 | 45 | 22,897,047 | 45 | 20,980,564 (91.6) |
| 100 μM BPA | B100-1 | 26,721,278 | 45 | 24,216,152 | 45 | 23,354,057 (96.4) |
|  | B100-2 | 29,559,279 | 45 | 27,586,083 | 45 | 26,557,122 (96.3) |
|  | B100-3 | 25,756,143 | 45 | 24,099,088 | 45 | 23,197,782 (96.3) |

**Supplementary table 4.** Differentially expressed genes (DEGs) downregulated between mouse SSCs treated with 100 μM BPA and 0 μM BPA

| **Gene symbol** | **Ensembl Gene** | **Entrez_id** | **logFC** | ***P-value*** | **FDR** |
| --- | --- | --- | --- | --- | --- |
| IL13RA2 | ENSMUSG00000031289 | 16165 | -6.63 | 7.0.E-08 | 1.3.E-04 |
| VNN3 | ENSMUSG00000020010 | 26464 | -6.55 | 2.4.E-04 | 2.9.E-02 |
| CXCL5 | ENSMUSG00000029371 | 20311 | -6.28 | 6.1.E-06 | 2.4.E-03 |
| *GM10309* | ENSMUSG00000071036 |  | -5.82 | 3.8.E-04 | 3.6.E-02 |
| NOS2 | ENSMUSG00000020826 | 18126 | -5.68 | 2.9.E-05 | 7.7.E-03 |
| GM7336 | ENSMUSG00000078636 |  | -5.26 | 2.3.E-06 | 1.3.E-03 |
| GM2606 | ENSMUSG00000078300 |  | -4.61 | 6.7.E-05 | 1.4.E-02 |
| TNFRSF9 | ENSMUSG00000028965 | 21942 | -4.28 | 1.1.E-04 | 1.9.E-02 |
| 2610528A11RIK | ENSMUSG00000096001 | 70045 | -4.27 | 5.4.E-04 | 4.4.E-02 |
| ADCYAP1 | ENSMUSG00000024256 | 11516 | -4.02 | 9.0.E-05 | 1.7.E-02 |
| BST1 | ENSMUSG00000029082 | 12182 | -3.95 | 5.6.E-05 | 1.3.E-02 |
| VWC2L | ENSMUSG00000045648 | 320460 | -3.83 | 6.5.E-05 | 1.4.E-02 |
| CPN2 | ENSMUSG00000023176 | 71756 | -3.65 | 6.0.E-04 | 4.5.E-02 |
| HP | ENSMUSG00000031722 | 15439 | -3.63 | 2.8.E-05 | 7.7.E-03 |
| GM47996 | ENSMUSG00000114591 |  | -3.61 | 3.5.E-04 | 3.5.E-02 |
| GM15987 | ENSMUSG00000085786 |  | -3.50 | 7.2.E-04 | 5.0.E-02 |
| RARRES2 | ENSMUSG00000009281 | 71660 | -3.47 | 6.4.E-06 | 2.5.E-03 |
| SLFN4 | ENSMUSG00000000204 | 20558 | -3.40 | 1.5.E-04 | 2.2.E-02 |
| KCNA1 | ENSMUSG00000047976 | 16485 | -3.37 | 2.6.E-05 | 7.4.E-03 |
| A2M | ENSMUSG00000030111 | 232345 | -3.30 | 5.4.E-05 | 1.3.E-02 |
| GM30117 | ENSMUSG00000117501 |  | -3.22 | 1.7.E-04 | 2.3.E-02 |
| CHIL1 | ENSMUSG00000064246 | 12654 | -3.08 | 9.1.E-06 | 3.4.E-03 |
| SLC15A2 | ENSMUSG00000022899 | 57738 | -3.06 | 4.6.E-04 | 4.1.E-02 |
| C1QTNF7 | ENSMUSG00000061535 | 109323 | -3.06 | 2.7.E-04 | 3.1.E-02 |
| GM36107 | ENSMUSG00000115439 |  | -3.02 | 1.3.E-04 | 2.0.E-02 |
| GM30717 | ENSMUSG00000108814 |  | -2.81 | 1.6.E-04 | 2.3.E-02 |
| GM8531 | ENSMUSG00000101085 |  | -2.79 | 4.8.E-04 | 4.1.E-02 |
| GM43185 | ENSMUSG00000104621 |  | -2.74 | 3.9.E-04 | 3.7.E-02 |
| GM41290 | ENSMUSG00000115428 |  | -2.71 | 6.7.E-04 | 4.8.E-02 |
| ASPN | ENSMUSG00000021388 | 66695 | -2.62 | 2.0.E-07 | 2.4.E-04 |
| FOXA2 | ENSMUSG00000037025 | 15376 | -2.48 | 4.1.E-06 | 1.9.E-03 |
| IL6 | ENSMUSG00000025746 | 16193 | -2.48 | 4.5.E-04 | 4.0.E-02 |
| STEAP4 | ENSMUSG00000012428 | 117167 | -2.48 | 3.0.E-04 | 3.2.E-02 |
| CCN5 | ENSMUSG00000027656 | 22403 | -2.44 | 6.4.E-05 | 1.4.E-02 |
| HPSE | ENSMUSG00000035273 | 15442 | -2.41 | 2.9.E-04 | 3.1.E-02 |
| MIR883B | ENSMUSG00000078024 |  | -2.31 | 9.5.E-05 | 1.7.E-02 |
| GM47322 | ENSMUSG00000113789 |  | -2.31 | 1.7.E-04 | 2.3.E-02 |
| COL12A1 | ENSMUSG00000032332 | 12816 | -2.30 | 3.2.E-04 | 3.3.E-02 |
| TNFSF11 | ENSMUSG00000022015 | 21943 | -2.29 | 1.6.E-04 | 2.3.E-02 |
| RASGRF1 | ENSMUSG00000032356 | 19417 | -2.24 | 2.3.E-05 | 7.1.E-03 |
| GNGT2 | ENSMUSG00000038811 | 14710 | -2.20 | 2.5.E-05 | 7.3.E-03 |
| MFAP5 | ENSMUSG00000030116 | 50530 | -2.19 | 2.0.E-05 | 6.4.E-03 |
| MDH1B | ENSMUSG00000025963 | 76668 | -2.19 | 6.4.E-04 | 4.7.E-02 |
| VSX2 | ENSMUSG00000021239 | 12677 | -2.17 | 7.2.E-06 | 2.8.E-03 |
| HAS2 | ENSMUSG00000022367 | 15117 | -2.14 | 6.2.E-04 | 4.6.E-02 |
| 4930470P17RIK | ENSMUSG00000043583 |  | -2.08 | 4.5.E-04 | 4.0.E-02 |
| GM8483 | ENSMUSG00000114737 |  | -2.07 | 5.4.E-04 | 4.4.E-02 |
| SLC2A3 | ENSMUSG00000003153 | 20527 | -2.06 | 1.8.E-06 | 1.2.E-03 |
| CFAP44 | ENSMUSG00000071550 | 212517 | -2.06 | 4.3.E-04 | 3.9.E-02 |
| DIO1 | ENSMUSG00000034785 | 13370 | -2.00 | 3.0.E-04 | 3.2.E-02 |
| GM38146 | ENSMUSG00000103163 |  | -1.95 | 1.6.E-04 | 2.3.E-02 |
| OVGP1 | ENSMUSG00000074340 | 12659 | -1.94 | 2.4.E-04 | 2.9.E-02 |
| GM43366 | ENSMUSG00000104665 |  | -1.88 | 5.6.E-04 | 4.4.E-02 |
| GM42372 | ENSMUSG00000108348 | 105247240 | -1.87 | 5.6.E-04 | 4.4.E-02 |
| PLAC8 | ENSMUSG00000029322 | 231507 | -1.84 | 1.9.E-04 | 2.5.E-02 |
| ANKRD37 | ENSMUSG00000050914 | 654824 | -1.84 | 7.0.E-04 | 5.0.E-02 |
| LRRC15 | ENSMUSG00000052316 | 74488 | -1.82 | 1.2.E-04 | 2.0.E-02 |
| SCN2B | ENSMUSG00000070304 | 72821 | -1.78 | 5.2.E-04 | 4.3.E-02 |
| PNMAL1 | ENSMUSG00000041141 | 71691 | -1.77 | 1.3.E-04 | 2.0.E-02 |
| NDRG2 | ENSMUSG00000004558 | 29811 | -1.73 | 4.4.E-04 | 3.9.E-02 |
| SEZ6L | ENSMUSG00000058153 | 56747 | -1.72 | 1.0.E-04 | 1.8.E-02 |
| COL5A3 | ENSMUSG00000004098 | 53867 | -1.60 | 2.7.E-04 | 3.0.E-02 |
| CELF3 | ENSMUSG00000028137 | 78784 | -1.58 | 3.4.E-04 | 3.4.E-02 |
| FRMD4B | ENSMUSG00000030064 | 232288 | -1.58 | 3.5.E-04 | 3.5.E-02 |
| MIRG | ENSMUSG00000097391 |  | -1.57 | 5.0.E-04 | 4.2.E-02 |
| PRSS8 | ENSMUSG00000030800 | 76560 | -1.56 | 4.1.E-04 | 3.8.E-02 |
| LY6G6E | ENSMUSG00000013766 | 70274 | -1.53 | 5.8.E-04 | 4.5.E-02 |
| IGFBP7 | ENSMUSG00000036256 | 29817 | -1.52 | 1.2.E-04 | 1.9.E-02 |
| SMARCA5-PS | ENSMUSG00000052912 |  | -1.50 | 1.2.E-04 | 1.9.E-02 |
| ARHGAP30 | ENSMUSG00000048865 | 226652 | -1.48 | 3.1.E-04 | 3.3.E-02 |
| GM37864 | ENSMUSG00000102401 |  | -1.47 | 6.7.E-04 | 4.8.E-02 |
| FTHL17-PS1 | ENSMUSG00000083714 |  | -1.46 | 4.5.E-04 | 4.0.E-02 |
| RPL35A-PS6 | ENSMUSG00000094664 |  | -1.40 | 1.5.E-04 | 2.2.E-02 |
| RASL11A | ENSMUSG00000029641 | 68895 | -1.40 | 1.6.E-04 | 2.3.E-02 |
| GPRIN2 | ENSMUSG00000071531 | 432839 | -1.37 | 6.7.E-04 | 4.8.E-02 |
| F2RL1 | ENSMUSG00000021678 | 14063 | -1.33 | 4.3.E-04 | 3.9.E-02 |
| H2BC1 | ENSMUSG00000050799 | 319177 | -1.25 | 3.5.E-04 | 3.5.E-02 |
| PDE4B | ENSMUSG00000028525 | 18578 | -1.24 | 4.9.E-05 | 1.2.E-02 |
| H2AC1 | ENSMUSG00000060081 | 319163 | -1.24 | 1.0.E-04 | 1.8.E-02 |
| SNHG11 | ENSMUSG00000044349 |  | -1.20 | 6.7.E-04 | 4.8.E-02 |
| EMB | ENSMUSG00000021728 | 13723 | -1.19 | 1.3.E-04 | 2.0.E-02 |
| TOPAZ1 | ENSMUSG00000094985 | 671232 | -1.15 | 8.4.E-05 | 1.7.E-02 |
| ZFP640 | ENSMUSG00000074830 |  | -1.10 | 2.0.E-04 | 2.6.E-02 |
| SLC38A4 | ENSMUSG00000022464 | 69354 | -1.06 | 9.9.E-05 | 1.8.E-02 |
| GM884 | ENSMUSG00000034239 | 380730 | -1.06 | 1.0.E-04 | 1.8.E-02 |
| NXF3 | ENSMUSG00000057000 | 245610 | -1.06 | 4.7.E-04 | 4.1.E-02 |
| PIWIL4 | ENSMUSG00000036912 | 330890 | -1.04 | 5.5.E-04 | 4.4.E-02 |

**Supplementary table 5.** Differentially expressed genes (DEGs) upregulated between mouse SSCs treated with 100 μM BPA and 0 μM dosage.

| **Gene symbol** | **Ensembl Gene** | **Entrez_id** | **logFC** | ***P-value*** | **FDR** |
| --- | --- | --- | --- | --- | --- |
| ALDH2 | ENSMUSG00000029455 | 11669 | 1.02 | 2.6.E-04 | 3.0.E-02 |
| CHST11 | ENSMUSG00000034612 | 58250 | 1.02 | 7.2.E-04 | 5.0.E-02 |
| GPD2 | ENSMUSG00000026827 | 14571 | 1.02 | 7.1.E-04 | 5.0.E-02 |
| ANTXR2 | ENSMUSG00000029338 | 71914 | 1.03 | 1.4.E-04 | 2.1.E-02 |
| MXRA7 | ENSMUSG00000020814 | 67622 | 1.03 | 2.9.E-04 | 3.1.E-02 |
| SEPTIN9 | ENSMUSG00000059248 | 53860 | 1.04 | 1.6.E-04 | 2.3.E-02 |
| NDUFA8 | ENSMUSG00000026895 | 68375 | 1.05 | 5.6.E-04 | 4.4.E-02 |
| PHLDA3 | ENSMUSG00000041801 | 27280 | 1.05 | 2.7.E-04 | 3.0.E-02 |
| GPRC5B | ENSMUSG00000008734 | 64297 | 1.05 | 5.9.E-04 | 4.5.E-02 |
| SCAMP2 | ENSMUSG00000040188 | 24044 | 1.06 | 1.8.E-04 | 2.4.E-02 |
| ACTG1 | ENSMUSG00000062825 | 11465 | 1.06 | 3.8.E-04 | 3.7.E-02 |
| DIPK1A | ENSMUSG00000029270 | 67266 | 1.07 | 6.1.E-05 | 1.3.E-02 |
| NCEH1 | ENSMUSG00000027698 | 320024 | 1.07 | 1.2.E-04 | 2.0.E-02 |
| MAN2A2 | ENSMUSG00000038886 | 140481 | 1.08 | 2.7.E-04 | 3.0.E-02 |
| GAS1 | ENSMUSG00000052957 | 14451 | 1.08 | 5.7.E-05 | 1.3.E-02 |
| PID1 | ENSMUSG00000045658 | 98496 | 1.09 | 6.3.E-04 | 4.7.E-02 |
| SCRN1 | ENSMUSG00000019124 | 69938 | 1.11 | 5.9.E-05 | 1.3.E-02 |
| NEU1 | ENSMUSG00000007038 | 18010 | 1.12 | 9.0.E-05 | 1.7.E-02 |
| ACSL5 | ENSMUSG00000024981 | 433256 | 1.12 | 4.1.E-04 | 3.8.E-02 |
| UNC5C | ENSMUSG00000059921 | 22253 | 1.13 | 3.7.E-04 | 3.6.E-02 |
| F3 | ENSMUSG00000028128 | 14066 | 1.13 | 3.9.E-04 | 3.7.E-02 |
| EDA2R | ENSMUSG00000034457 | 245527 | 1.13 | 3.3.E-04 | 3.3.E-02 |
| PSRC1 | ENSMUSG00000068744 | 56742 | 1.13 | 1.1.E-04 | 1.9.E-02 |
| SH3PXD2B | ENSMUSG00000040711 | 268396 | 1.14 | 3.1.E-04 | 3.3.E-02 |
| SORD | ENSMUSG00000027227 | 20322 | 1.15 | 1.4.E-04 | 2.1.E-02 |
| SLC25A5 | ENSMUSG00000016319 | 11740 | 1.15 | 2.6.E-04 | 3.0.E-02 |
| PEG3 | ENSMUSG00000002265 | 18616 | 1.15 | 2.2.E-04 | 2.7.E-02 |
| IDUA | ENSMUSG00000033540 | 15932 | 1.15 | 7.2.E-04 | 5.0.E-02 |
| RAB7 | ENSMUSG00000079477 | 19349 | 1.16 | 5.7.E-04 | 4.5.E-02 |
| IPO13 | ENSMUSG00000033365 | 230673 | 1.16 | 1.3.E-04 | 2.0.E-02 |
| NCALD | ENSMUSG00000051359 | 52589 | 1.16 | 4.4.E-04 | 4.0.E-02 |
| TRPS1 | ENSMUSG00000038679 | 83925 | 1.16 | 5.2.E-04 | 4.3.E-02 |
| DPP7 | ENSMUSG00000026958 | 83768 | 1.17 | 1.8.E-05 | 6.0.E-03 |
| FAM214B | ENSMUSG00000036002 | 230088 | 1.18 | 1.9.E-04 | 2.5.E-02 |
| COLEC12 | ENSMUSG00000036103 | 140792 | 1.18 | 9.4.E-05 | 1.7.E-02 |
| OAF | ENSMUSG00000032014 | 102644 | 1.18 | 2.1.E-04 | 2.6.E-02 |
| ARL8A | ENSMUSG00000026426 | 68724 | 1.18 | 2.0.E-04 | 2.6.E-02 |
| SULF2 | ENSMUSG00000006800 | 72043 | 1.18 | 1.6.E-04 | 2.3.E-02 |
| FAM20C | ENSMUSG00000025854 | 80752 | 1.18 | 4.7.E-04 | 4.1.E-02 |
| SLC48A1 | ENSMUSG00000081534 | 67739 | 1.18 | 8.9.E-05 | 1.7.E-02 |
| FEZ1 | ENSMUSG00000032118 | 235180 | 1.19 | 3.8.E-04 | 3.6.E-02 |
| ARHGAP24 | ENSMUSG00000057315 | 231532 | 1.19 | 5.0.E-06 | 2.1.E-03 |
| ORAI2 | ENSMUSG00000039747 | 269717 | 1.19 | 2.7.E-05 | 7.5.E-03 |
| TAP2 | ENSMUSG00000024339 | 21355 | 1.19 | 5.2.E-04 | 4.3.E-02 |
| TNFRSF12A | ENSMUSG00000023905 | 27279 | 1.20 | 3.9.E-04 | 3.7.E-02 |
| PML | ENSMUSG00000036986 | 18854 | 1.20 | 4.9.E-04 | 4.2.E-02 |
| PARD6G | ENSMUSG00000056214 | 93737 | 1.20 | 4.9.E-04 | 4.2.E-02 |
| CSGALNACT1 | ENSMUSG00000036356 | 234356 | 1.22 | 2.5.E-05 | 7.2.E-03 |
| APCDD1 | ENSMUSG00000071847 | 494504 | 1.22 | 1.1.E-04 | 1.9.E-02 |
| MMP14 | ENSMUSG00000000957 | 17387 | 1.22 | 1.3.E-04 | 2.0.E-02 |
| TMEM63B | ENSMUSG00000036026 | 224807 | 1.23 | 5.5.E-05 | 1.3.E-02 |
| ZFP365 | ENSMUSG00000037855 | 216049 | 1.23 | 6.0.E-05 | 1.3.E-02 |
| NPTXR | ENSMUSG00000022421 | 73340 | 1.24 | 2.6.E-04 | 3.0.E-02 |
| MMP2 | ENSMUSG00000031740 | 17390 | 1.25 | 1.3.E-05 | 4.5.E-03 |
| ST6GALNAC4 | ENSMUSG00000079442 | 20448 | 1.25 | 2.1.E-04 | 2.6.E-02 |
| FTH1 | ENSMUSG00000024661 | 14319 | 1.26 | 6.7.E-04 | 4.8.E-02 |
| SSPN | ENSMUSG00000030255 | 16651 | 1.26 | 2.7.E-05 | 7.5.E-03 |
| GNS | ENSMUSG00000034707 | 75612 | 1.26 | 8.7.E-05 | 1.7.E-02 |
| AGPAT3 | ENSMUSG00000001211 | 28169 | 1.27 | 9.2.E-06 | 3.4.E-03 |
| MYO5A | ENSMUSG00000034593 | 17918 | 1.27 | 1.3.E-06 | 9.0.E-04 |
| H19 | ENSMUSG00000000031 |  | 1.27 | 6.2.E-04 | 4.7.E-02 |
| 5031439G07RIK | ENSMUSG00000036046 | 223739 | 1.27 | 3.1.E-05 | 8.2.E-03 |
| OSR1 | ENSMUSG00000048387 | 23967 | 1.27 | 1.7.E-04 | 2.3.E-02 |
| HMGA1B | ENSMUSG00000078249 | 111241 | 1.28 | 2.1.E-04 | 2.6.E-02 |
| MFAP3L | ENSMUSG00000031647 | 71306 | 1.29 | 2.8.E-04 | 3.1.E-02 |
| PLP1 | ENSMUSG00000031425 | 18823 | 1.29 | 6.1.E-04 | 4.6.E-02 |
| HVCN1 | ENSMUSG00000064267 | 74096 | 1.30 | 2.4.E-05 | 7.2.E-03 |
| CDH13 | ENSMUSG00000031841 | 12554 | 1.30 | 5.1.E-04 | 4.3.E-02 |
| MAOA | ENSMUSG00000025037 | 17161 | 1.31 | 4.5.E-05 | 1.1.E-02 |
| ATF5 | ENSMUSG00000038539 | 107503 | 1.32 | 1.1.E-04 | 1.9.E-02 |
| STEAP3 | ENSMUSG00000026389 | 68428 | 1.33 | 5.5.E-05 | 1.3.E-02 |
| RGMB | ENSMUSG00000048027 | 68799 | 1.36 | 4.7.E-06 | 2.1.E-03 |
| TIMP2 | ENSMUSG00000017466 | 21858 | 1.37 | 4.4.E-05 | 1.1.E-02 |
| IRGM1 | ENSMUSG00000046879 | 15944 | 1.37 | 1.5.E-04 | 2.2.E-02 |
| IER2 | ENSMUSG00000053560 | 15936 | 1.38 | 5.3.E-04 | 4.4.E-02 |
| SRM | ENSMUSG00000006442 | 20810 | 1.40 | 3.8.E-04 | 3.7.E-02 |
| C030006K11RIK | ENSMUSG00000116138 | 223665 | 1.41 | 3.2.E-04 | 3.3.E-02 |
| IFIT3 | ENSMUSG00000074896 | 15959 | 1.42 | 7.7.E-05 | 1.6.E-02 |
| SPRY2 | ENSMUSG00000022114 | 24064 | 1.42 | 9.4.E-06 | 3.4.E-03 |
| NRP1 | ENSMUSG00000025810 | 18186 | 1.42 | 2.9.E-06 | 1.4.E-03 |
| PRRG4 | ENSMUSG00000027171 | 228413 | 1.43 | 5.9.E-04 | 4.5.E-02 |
| ST6GAL1 | ENSMUSG00000022885 | 20440 | 1.43 | 2.2.E-05 | 7.0.E-03 |
| TSHZ1 | ENSMUSG00000046982 | 110796 | 1.44 | 4.5.E-05 | 1.1.E-02 |
| GATA4 | ENSMUSG00000021944 | 14463 | 1.44 | 5.9.E-04 | 4.5.E-02 |
| GAS7 | ENSMUSG00000033066 | 14457 | 1.45 | 1.7.E-06 | 1.1.E-03 |
| LDHB | ENSMUSG00000030246 | 16832 | 1.46 | 4.9.E-06 | 2.1.E-03 |
| UAP1L1 | ENSMUSG00000026956 | 227620 | 1.46 | 2.4.E-07 | 2.8.E-04 |
| FRMD4A | ENSMUSG00000026657 | 209630 | 1.47 | 6.6.E-05 | 1.4.E-02 |
| CDH26 | ENSMUSG00000039155 | 381409 | 1.47 | 5.5.E-04 | 4.4.E-02 |
| EPHA7 | ENSMUSG00000028289 | 13841 | 1.47 | 1.1.E-05 | 3.9.E-03 |
| L3MBTL3 | ENSMUSG00000039089 | 237339 | 1.49 | 3.3.E-04 | 3.3.E-02 |
| NR2F1 | ENSMUSG00000069171 | 13865 | 1.50 | 4.3.E-05 | 1.1.E-02 |
| CCDC107 | ENSMUSG00000028461 | 622404 | 1.50 | 2.0.E-04 | 2.6.E-02 |
| SCN1B | ENSMUSG00000019194 | 20266 | 1.50 | 1.9.E-06 | 1.2.E-03 |
| CAPN6 | ENSMUSG00000067276 | 12338 | 1.50 | 2.6.E-07 | 2.8.E-04 |
| PLXNA2 | ENSMUSG00000026640 | 18845 | 1.50 | 1.5.E-07 | 2.2.E-04 |
| MEF2C | ENSMUSG00000005583 | 17260 | 1.50 | 2.8.E-04 | 3.1.E-02 |
| GLMP | ENSMUSG00000001418 | 56700 | 1.51 | 1.1.E-04 | 1.9.E-02 |
| ATP6V1C1 | ENSMUSG00000022295 | 66335 | 1.51 | 6.7.E-04 | 4.8.E-02 |
| LAPTM4B | ENSMUSG00000022257 | 114128 | 1.53 | 2.4.E-06 | 1.3.E-03 |
| EPS8 | ENSMUSG00000015766 | 13860 | 1.53 | 1.8.E-05 | 5.9.E-03 |
| GM3362 | ENSMUSG00000085442 |  | 1.53 | 3.0.E-07 | 3.0.E-04 |
| GUSB | ENSMUSG00000025534 | 110006 | 1.55 | 6.0.E-06 | 2.4.E-03 |
| SLC6A8 | ENSMUSG00000019558 | 102857 | 1.56 | 6.1.E-08 | 1.3.E-04 |
| EFNB2 | ENSMUSG00000001300 | 13642 | 1.58 | 2.0.E-04 | 2.6.E-02 |
| CMTM3 | ENSMUSG00000031875 | 68119 | 1.59 | 6.6.E-08 | 1.3.E-04 |
| SH3RF3 | ENSMUSG00000037990 | 237353 | 1.59 | 2.4.E-04 | 2.9.E-02 |
| GASK1B | ENSMUSG00000027955 | 68659 | 1.59 | 9.6.E-07 | 7.1.E-04 |
| BVHT | ENSMUSG00000098098 |  | 1.60 | 1.5.E-04 | 2.2.E-02 |
| CLDN1 | ENSMUSG00000022512 | 12737 | 1.62 | 7.0.E-04 | 5.0.E-02 |
| CELA1 | ENSMUSG00000023031 | 109901 | 1.65 | 3.7.E-04 | 3.6.E-02 |
| NAGLU | ENSMUSG00000001751 | 27419 | 1.65 | 3.6.E-07 | 3.0.E-04 |
| PCDHGC3 | ENSMUSG00000102918 | 93706 | 1.68 | 7.0.E-07 | 5.4.E-04 |
| ETV1 | ENSMUSG00000004151 | 14009 | 1.68 | 4.0.E-06 | 1.9.E-03 |
| GJA1 | ENSMUSG00000050953 | 14609 | 1.68 | 6.3.E-04 | 4.7.E-02 |
| RPL3-PS2 | ENSMUSG00000084131 |  | 1.68 | 2.1.E-05 | 6.6.E-03 |
| ZADH2 | ENSMUSG00000049090 | 225791 | 1.69 | 9.1.E-05 | 1.7.E-02 |
| FRRS1 | ENSMUSG00000033386 | 20321 | 1.69 | 1.7.E-05 | 5.9.E-03 |
| CCND1 | ENSMUSG00000070348 | 12443 | 1.71 | 1.0.E-08 | 2.6.E-05 |
| MEGF10 | ENSMUSG00000024593 | 70417 | 1.73 | 5.9.E-04 | 4.5.E-02 |
| PCDH17 | ENSMUSG00000035566 | 219228 | 1.74 | 1.6.E-04 | 2.3.E-02 |
| GM11353 | ENSMUSG00000060198 |  | 1.75 | 2.3.E-04 | 2.9.E-02 |
| GM23935 | ENSMUSG00000076258 |  | 1.80 | 2.5.E-04 | 3.0.E-02 |
| SEMA6A | ENSMUSG00000019647 | 20358 | 1.81 | 7.1.E-05 | 1.5.E-02 |
| PLAU | ENSMUSG00000021822 | 18792 | 1.84 | 3.5.E-07 | 3.0.E-04 |
| SEMA5A | ENSMUSG00000022231 | 20356 | 1.84 | 2.4.E-06 | 1.3.E-03 |
| GM10132 | ENSMUSG00000063556 |  | 1.85 | 7.1.E-04 | 5.0.E-02 |
| SLC35F1 | ENSMUSG00000038602 | 215085 | 1.87 | 8.5.E-05 | 1.7.E-02 |
| SLC8A3 | ENSMUSG00000079055 | 110893 | 1.87 | 5.6.E-04 | 4.4.E-02 |
| FGF1 | ENSMUSG00000036585 | 14164 | 1.88 | 2.3.E-06 | 1.3.E-03 |
| LRRC4B | ENSMUSG00000047085 | 272381 | 1.89 | 9.2.E-05 | 1.7.E-02 |
| ANK | ENSMUSG00000022265 | 11732 | 1.89 | 2.6.E-11 | 1.6.E-07 |
| SNAI2 | ENSMUSG00000022676 | 20583 | 1.91 | 1.9.E-05 | 6.1.E-03 |
| GM7432 | ENSMUSG00000109610 |  | 1.93 | 2.9.E-04 | 3.2.E-02 |
| IFIT3B | ENSMUSG00000062488 | 667370 | 1.94 | 1.1.E-06 | 7.8.E-04 |
| OLFR1314 | ENSMUSG00000074945 | 258442 | 1.95 | 4.1.E-06 | 1.9.E-03 |
| HMGA2 | ENSMUSG00000056758 | 15364 | 1.96 | 8.9.E-08 | 1.5.E-04 |
| CPE | ENSMUSG00000037852 | 12876 | 2.00 | 1.7.E-07 | 2.3.E-04 |
| ITGA8 | ENSMUSG00000026768 | 241226 | 2.01 | 1.9.E-10 | 7.1.E-07 |
| SPARCL1 | ENSMUSG00000029309 | 13602 | 2.05 | 2.1.E-06 | 1.3.E-03 |
| CST6 | ENSMUSG00000024846 | 73720 | 2.14 | 4.8.E-07 | 3.9.E-04 |
| CD200 | ENSMUSG00000022661 | 17470 | 2.17 | 2.6.E-06 | 1.3.E-03 |
| SORCS2 | ENSMUSG00000029093 | 81840 | 2.18 | 4.3.E-09 | 1.3.E-05 |
| EVI2A | ENSMUSG00000078771 | 14017 | 2.19 | 3.2.E-07 | 3.0.E-04 |
| GM5454 | ENSMUSG00000047643 |  | 2.27 | 3.1.E-05 | 8.1.E-03 |
| CAMK2N2 | ENSMUSG00000051146 | 73047 | 2.27 | 1.1.E-04 | 1.9.E-02 |
| PPARGC1A | ENSMUSG00000029167 | 19017 | 2.29 | 1.2.E-10 | 5.7.E-07 |
| SLC7A11 | ENSMUSG00000027737 | 26570 | 2.31 | 3.1.E-04 | 3.3.E-02 |
| GM10362 | ENSMUSG00000116174 |  | 2.46 | 4.4.E-05 | 1.1.E-02 |
| RN7S2 | ENSMUSG00000099250 |  | 2.49 | 2.6.E-04 | 3.0.E-02 |
| RAB7-PS1 | ENSMUSG00000111686 |  | 2.50 | 5.0.E-04 | 4.2.E-02 |
| ARHGAP6 | ENSMUSG00000031355 | 11856 | 2.70 | 1.0.E-07 | 1.6.E-04 |
| AHNAK2 | ENSMUSG00000072812 | 100041194 | 2.88 | 2.1.E-11 | 1.6.E-07 |
| FTL2-PS | ENSMUSG00000082062 |  | 3.15 | 3.1.E-04 | 3.3.E-02 |
| MGP | ENSMUSG00000030218 | 17313 | 3.95 | 1.4.E-17 | 2.6.E-13 |
| GM42613 | ENSMUSG00000106575 |  | 4.16 | 3.9.E-04 | 3.7.E-02 |
| GM14461 | ENSMUSG00000054510 | 329436 | 4.26 | 2.9.E-06 | 1.4.E-03 |
| ATP6V0D2 | ENSMUSG00000028238 | 242341 | 6.04 | 1.0.E-04 | 1.8.E-02 |
| GM10231 | ENSMUSG00000068120 |  | 6.18 | 3.3.E-07 | 3.0.E-04 |
| RTL8A | ENSMUSG00000067925 | 66158 | 6.66 | 9.0.E-05 | 1.7.E-02 |

**Supplementary table 6.** GO and KEGG pathway enrichment analyses of DEGs in SSCs treated with 100 μM BPA

| **Category** | **Term** | **Description** | **%** | ***P-value*** | **Fold Enrichment** | **Benjamini** |
| --- | --- | --- | --- | --- | --- | --- |
| BP | GO:0008285 | negative regulation of cell proliferation | 6.57 | 1.60.E-04 | 3.56.E+00 | 1.11.E-01 |
| BP | GO:0007275 | multicellular organism development | 11.27 | 3.24.E-04 | 2.28.E+00 | 1.12.E-01 |
| BP | GO:0045944 | positive regulation of transcription from RNA polymerase II promoter | 10.80 | 5.09.E-04 | 2.26.E+00 | 1.18.E-01 |
| BP | GO:0005975 | carbohydrate metabolic process | 4.69 | 2.66.E-04 | 4.74.E+00 | 1.23.E-01 |
| BP | GO:0045666 | positive regulation of neuron differentiation | 3.29 | 6.47.E-04 | 6.64.E+00 | 1.27.E-01 |
| BP | GO:0051897 | positive regulation of protein kinase B signaling | 3.29 | 4.70.E-04 | 7.05.E+00 | 1.29.E-01 |
| BP | GO:0045165 | cell fate commitment | 2.82 | 8.33.E-04 | 8.15.E+00 | 1.42.E-01 |
| BP | GO:0030335 | positive regulation of cell migration | 4.23 | 1.14.E-03 | 4.33.E+00 | 1.70.E-01 |
| BP | GO:0007155 | cell adhesion | 6.57 | 1.43.E-03 | 2.82.E+00 | 1.90.E-01 |
| BP | GO:0007411 | axon guidance | 4.23 | 1.45.E-04 | 5.90.E+00 | 1.92.E-01 |
| BP | GO:0042474 | middle ear morphogenesis | 1.88 | 1.79.E-03 | 1.63.E+01 | 1.98.E-01 |
| BP | GO:0007399 | nervous system development | 5.63 | 1.71.E-03 | 3.11.E+00 | 2.04.E-01 |
| BP | GO:0010976 | positive regulation of neuron projection development | 3.29 | 2.90.E-03 | 4.96.E+00 | 2.23.E-01 |
| BP | GO:0060371 | regulation of atrial cardiac muscle cell membrane depolarization | 1.41 | 2.77.E-03 | 3.67.E+01 | 2.25.E-01 |
| BP | GO:0030154 | cell differentiation | 8.45 | 2.60.E-03 | 2.26.E+00 | 2.26.E-01 |
| BP | GO:0042127 | regulation of cell proliferation | 4.23 | 2.30.E-03 | 3.88.E+00 | 2.30.E-01 |
| BP | GO:0035987 | endodermal cell differentiation | 1.88 | 2.53.E-03 | 1.45.E+01 | 2.34.E-01 |
| BP | GO:0071526 | semaphorin-plexin signaling pathway | 1.88 | 4.92.E-03 | 1.15.E+01 | 3.32.E-01 |
| BP | GO:0048754 | branching morphogenesis of an epithelial tube | 1.88 | 5.78.E-03 | 1.09.E+01 | 3.62.E-01 |
| BP | GO:0045909 | positive regulation of vasodilation | 1.88 | 6.73.E-03 | 1.03.E+01 | 3.92.E-01 |
| BP | GO:0046426 | negative regulation of JAK-STAT cascade | 1.88 | 9.49.E-03 | 9.09.E+00 | 4.06.E-01 |
| BP | GO:0006954 | inflammatory response | 4.69 | 8.80.E-03 | 2.84.E+00 | 4.06.E-01 |
| BP | GO:0090162 | establishment of epithelial cell polarity | 1.41 | 9.91.E-03 | 1.95.E+01 | 4.08.E-01 |
| BP | GO:0055114 | oxidation-reduction process | 7.04 | 9.39.E-03 | 2.17.E+00 | 4.14.E-01 |
| BP | GO:0008152 | metabolic process | 5.63 | 7.95.E-03 | 2.53.E+00 | 4.14.E-01 |
| BP | GO:0030324 | lung development | 2.82 | 8.79.E-03 | 4.73.E+00 | 4.18.E-01 |
| BP | GO:0001525 | angiogenesis | 3.76 | 1.15.E-02 | 3.27.E+00 | 4.22.E-01 |
| BP | GO:0048771 | tissue remodeling | 1.41 | 8.65.E-03 | 2.09.E+01 | 4.27.E-01 |
| BP | GO:0045766 | positive regulation of angiogenesis | 2.82 | 7.95.E-03 | 4.85.E+00 | 4.29.E-01 |
| BP | GO:0002062 | chondrocyte differentiation | 1.88 | 1.14.E-02 | 8.50.E+00 | 4.31.E-01 |
| BP | GO:2000310 | regulation of N-methyl-D-aspartate selective glutamate receptor activity | 1.41 | 1.13.E-02 | 1.83.E+01 | 4.37.E-01 |
| BP | GO:0070374 | positive regulation of ERK1 and ERK2 cascade | 3.29 | 1.26.E-02 | 3.64.E+00 | 4.43.E-01 |
| BP | GO:0006915 | apoptotic process | 6.10 | 1.39.E-02 | 2.23.E+00 | 4.66.E-01 |
| BP | GO:0019221 | cytokine-mediated signaling pathway | 2.82 | 1.69.E-02 | 4.02.E+00 | 5.22.E-01 |
| BP | GO:0007507 | heart development | 3.76 | 1.76.E-02 | 3.00.E+00 | 5.27.E-01 |
| BP | GO:0030500 | regulation of bone mineralization | 1.41 | 1.90.E-02 | 1.40.E+01 | 5.45.E-01 |
| BP | GO:0098781 | ncRNA transcription | 0.94 | 2.02.E-02 | 9.77.E+01 | 5.57.E-01 |
| BP | GO:0010628 | positive regulation of gene expression | 4.69 | 2.10.E-02 | 2.45.E+00 | 5.60.E-01 |
| BP | GO:0008284 | positive regulation of cell proliferation | 5.63 | 2.31.E-02 | 2.16.E+00 | 5.87.E-01 |
| BP | GO:0010575 | positive regulation of vascular endothelial growth factor production | 1.41 | 2.45.E-02 | 1.22.E+01 | 6.00.E-01 |
| BP | GO:0009612 | response to mechanical stimulus | 1.88 | 2.64.E-02 | 6.21.E+00 | 6.08.E-01 |
| BP | GO:0071356 | cellular response to tumor necrosis factor | 2.35 | 2.60.E-02 | 4.44.E+00 | 6.13.E-01 |
| BP | GO:0042476 | odontogenesis | 1.41 | 3.06.E-02 | 1.09.E+01 | 6.23.E-01 |
| BP | GO:0090402 | oncogene-induced cell senescence | 0.94 | 3.02.E-02 | 6.52.E+01 | 6.26.E-01 |
| BP | GO:0006810 | transport | 13.15 | 2.97.E-02 | 1.50.E+00 | 6.28.E-01 |
| BP | GO:0048843 | negative regulation of axon extension involved in axon guidance | 1.41 | 2.85.E-02 | 1.13.E+01 | 6.29.E-01 |
| BP | GO:0042733 | embryonic digit morphogenesis | 1.88 | 2.97.E-02 | 5.92.E+00 | 6.36.E-01 |
| BP | GO:0001958 | endochondral ossification | 1.41 | 3.72.E-02 | 9.77.E+00 | 6.80.E-01 |
| BP | GO:0001764 | neuron migration | 2.35 | 3.80.E-02 | 3.94.E+00 | 6.81.E-01 |
| BP | GO:0006814 | sodium ion transport | 2.35 | 3.80.E-02 | 3.94.E+00 | 6.81.E-01 |
| BP | GO:0006811 | ion transport | 5.63 | 3.69.E-02 | 2.01.E+00 | 6.85.E-01 |
| BP | GO:0001934 | positive regulation of protein phosphorylation | 2.82 | 4.01.E-02 | 3.19.E+00 | 6.87.E-01 |
| BP | GO:0043627 | response to estrogen | 1.88 | 4.11.E-02 | 5.21.E+00 | 6.88.E-01 |
| BP | GO:2000345 | regulation of hepatocyte proliferation | 0.94 | 4.01.E-02 | 4.89.E+01 | 6.93.E-01 |
| BP | GO:0097461 | ferric iron import into cell | 0.94 | 4.01.E-02 | 4.89.E+01 | 6.93.E-01 |
| BP | GO:0003104 | positive regulation of glomerular filtration | 0.94 | 4.01.E-02 | 4.89.E+01 | 6.93.E-01 |
| BP | GO:0035986 | senescence-associated heterochromatin focus assembly | 0.94 | 4.01.E-02 | 4.89.E+01 | 6.93.E-01 |
| BP | GO:0072606 | interleukin-8 secretion | 0.94 | 4.01.E-02 | 4.89.E+01 | 6.93.E-01 |
| BP | GO:0010644 | cell communication by electrical coupling | 0.94 | 4.01.E-02 | 4.89.E+01 | 6.93.E-01 |
| BP | GO:0050679 | positive regulation of epithelial cell proliferation | 1.88 | 4.24.E-02 | 5.14.E+00 | 6.94.E-01 |
| BP | GO:0071392 | cellular response to estradiol stimulus | 1.41 | 4.42.E-02 | 8.89.E+00 | 7.03.E-01 |
| BP | GO:0045444 | fat cell differentiation | 1.88 | 4.67.E-02 | 4.95.E+00 | 7.10.E-01 |
| BP | GO:0060612 | adipose tissue development | 1.41 | 4.67.E-02 | 8.62.E+00 | 7.16.E-01 |
| BP | GO:0072178 | nephric duct morphogenesis | 0.94 | 4.99.E-02 | 3.91.E+01 | 7.27.E-01 |
| BP | GO:0032642 | regulation of chemokine production | 0.94 | 4.99.E-02 | 3.91.E+01 | 7.27.E-01 |
| BP | GO:0070741 | response to interleukin 6 | 0.94 | 4.99.E-02 | 3.91.E+01 | 7.27.E-01 |
| BP | GO:2000987 | positive regulation of behavioral fear response | 0.94 | 4.99.E-02 | 3.91.E+01 | 7.27.E-01 |
| BP | GO:0010574 | regulation of vascular endothelial growth factor production | 0.94 | 4.99.E-02 | 3.91.E+01 | 7.27.E-01 |
| BP | GO:0046684 | response to pyrethroid | 0.94 | 4.99.E-02 | 3.91.E+01 | 7.27.E-01 |
| BP | GO:0035725 | sodium ion transmembrane transport | 1.41 | 5.18.E-02 | 8.15.E+00 | 7.29.E-01 |
| BP | GO:0051216 | cartilage development | 1.88 | 5.12.E-02 | 4.77.E+00 | 7.31.E-01 |
| BP | GO:0060021 | palate development | 1.88 | 5.59.E-02 | 4.60.E+00 | 7.39.E-01 |
| BP | GO:0030501 | positive regulation of bone mineralization | 1.41 | 5.44.E-02 | 7.92.E+00 | 7.41.E-01 |
| BP | GO:0043065 | positive regulation of apoptotic process | 3.76 | 5.58.E-02 | 2.33.E+00 | 7.45.E-01 |
| BP | GO:0014911 | positive regulation of smooth muscle cell migration | 1.41 | 5.97.E-02 | 7.52.E+00 | 7.52.E-01 |
| BP | GO:2001171 | positive regulation of ATP biosynthetic process | 0.94 | 5.95.E-02 | 3.26.E+01 | 7.57.E-01 |
| BP | GO:2001028 | positive regulation of endothelial cell chemotaxis | 0.94 | 5.95.E-02 | 3.26.E+01 | 7.57.E-01 |
| BP | GO:0086012 | membrane depolarization during cardiac muscle cell action potential | 0.94 | 5.95.E-02 | 3.26.E+01 | 7.57.E-01 |
| BP | GO:0009409 | response to cold | 1.41 | 6.24.E-02 | 7.33.E+00 | 7.63.E-01 |
| BP | GO:0043066 | negative regulation of apoptotic process | 5.16 | 6.43.E-02 | 1.90.E+00 | 7.68.E-01 |
| BP | GO:0043123 | positive regulation of I-kappaB kinase/NF-kappaB signaling | 2.35 | 6.59.E-02 | 3.28.E+00 | 7.72.E-01 |
| BP | GO:0007162 | negative regulation of cell adhesion | 1.41 | 6.80.E-02 | 6.98.E+00 | 7.78.E-01 |
| BP | GO:0048841 | regulation of axon extension involved in axon guidance | 0.94 | 6.91.E-02 | 2.79.E+01 | 7.79.E-01 |
| BP | GO:0060536 | cartilage morphogenesis | 0.94 | 6.91.E-02 | 2.79.E+01 | 7.79.E-01 |
| BP | GO:0015677 | copper ion import | 0.94 | 6.91.E-02 | 2.79.E+01 | 7.79.E-01 |
| BP | GO:0030204 | chondroitin sulfate metabolic process | 0.94 | 6.91.E-02 | 2.79.E+01 | 7.79.E-01 |
| BP | GO:0071864 | positive regulation of cell proliferation in bone marrow | 0.94 | 6.91.E-02 | 2.79.E+01 | 7.79.E-01 |
| BP | GO:0048842 | positive regulation of axon extension involved in axon guidance | 0.94 | 6.91.E-02 | 2.79.E+01 | 7.79.E-01 |
| BP | GO:0045893 | positive regulation of transcription, DNA-templated | 5.16 | 7.04.E-02 | 1.87.E+00 | 7.81.E-01 |
| BP | GO:0006469 | negative regulation of protein kinase activity | 1.88 | 7.29.E-02 | 4.12.E+00 | 7.87.E-01 |
| BP | GO:0036302 | atrioventricular canal development | 0.94 | 7.86.E-02 | 2.44.E+01 | 8.08.E-01 |
| BP | GO:2000543 | positive regulation of gastrulation | 0.94 | 7.86.E-02 | 2.44.E+01 | 8.08.E-01 |
| BP | GO:1902533 | positive regulation of intracellular signal transduction | 0.94 | 7.86.E-02 | 2.44.E+01 | 8.08.E-01 |
| BP | GO:0090005 | negative regulation of establishment of protein localization to plasma membrane | 0.94 | 7.86.E-02 | 2.44.E+01 | 8.08.E-01 |
| BP | GO:0043615 | astrocyte cell migration | 0.94 | 7.86.E-02 | 2.44.E+01 | 8.08.E-01 |
| BP | GO:0009725 | response to hormone | 1.41 | 8.57.E-02 | 6.11.E+00 | 8.28.E-01 |
| BP | GO:0007156 | homophilic cell adhesion via plasma membrane adhesion molecules | 2.35 | 8.52.E-02 | 3.00.E+00 | 8.30.E-01 |
| BP | GO:0045600 | positive regulation of fat cell differentiation | 1.41 | 8.88.E-02 | 5.98.E+00 | 8.31.E-01 |
| BP | GO:0048468 | cell development | 1.41 | 8.88.E-02 | 5.98.E+00 | 8.31.E-01 |
| BP | GO:0060290 | transdifferentiation | 0.94 | 8.80.E-02 | 2.17.E+01 | 8.32.E-01 |
| BP | GO:0035988 | chondrocyte proliferation | 0.94 | 8.80.E-02 | 2.17.E+01 | 8.32.E-01 |
| BP | GO:0042759 | long-chain fatty acid biosynthetic process | 0.94 | 8.80.E-02 | 2.17.E+01 | 8.32.E-01 |
| BP | GO:2000774 | positive regulation of cellular senescence | 0.94 | 8.80.E-02 | 2.17.E+01 | 8.32.E-01 |
| BP | GO:0030836 | positive regulation of actin filament depolymerization | 0.94 | 8.80.E-02 | 2.17.E+01 | 8.32.E-01 |
| BP | GO:0051607 | defense response to virus | 2.35 | 9.12.E-02 | 2.93.E+00 | 8.36.E-01 |
| BP | GO:0051091 | positive regulation of sequence-specific DNA binding transcription factor activity | 1.88 | 9.59.E-02 | 3.65.E+00 | 8.47.E-01 |
| BP | GO:0001942 | hair follicle development | 1.41 | 9.82.E-02 | 5.64.E+00 | 8.48.E-01 |
| BP | GO:0086002 | cardiac muscle cell action potential involved in contraction | 0.94 | 9.72.E-02 | 1.95.E+01 | 8.48.E-01 |
| BP | GO:0048703 | embryonic viscerocranium morphogenesis | 0.94 | 9.72.E-02 | 1.95.E+01 | 8.48.E-01 |
| BP | GO:2000810 | regulation of bicellular tight junction assembly | 0.94 | 9.72.E-02 | 1.95.E+01 | 8.48.E-01 |
| BP | GO:2000727 | positive regulation of cardiac muscle cell differentiation | 0.94 | 9.72.E-02 | 1.95.E+01 | 8.48.E-01 |
| BP | GO:0006032 | chitin catabolic process | 0.94 | 9.72.E-02 | 1.95.E+01 | 8.48.E-01 |
| BP | GO:0033598 | mammary gland epithelial cell proliferation | 0.94 | 9.72.E-02 | 1.95.E+01 | 8.48.E-01 |
| BP | GO:0009887 | organ morphogenesis | 1.88 | 1.00.E-01 | 3.59.E+00 | 8.49.E-01 |
| CC | GO:0016020 | membrane | 50.23 | 8.38.E-08 | 1.53.E+00 | 2.04.E-05 |
| CC | GO:0070062 | extracellular exosome | 24.88 | 1.08.E-06 | 1.98.E+00 | 1.31.E-04 |
| CC | GO:0005615 | extracellular space | 16.90 | 2.03.E-06 | 2.39.E+00 | 1.65.E-04 |
| CC | GO:0005764 | lysosome | 6.10 | 1.27.E-04 | 3.92.E+00 | 7.72.E-03 |
| CC | GO:0031012 | extracellular matrix | 5.63 | 1.85.E-04 | 4.07.E+00 | 8.95.E-03 |
| CC | GO:0005576 | extracellular region | 15.49 | 5.91.E-04 | 1.88.E+00 | 2.37.E-02 |
| CC | GO:0005887 | integral component of plasma membrane | 11.27 | 8.69.E-04 | 2.13.E+00 | 2.97.E-02 |
| CC | GO:0005765 | lysosomal membrane | 3.76 | 9.20.E-03 | 3.41.E+00 | 2.45.E-01 |
| CC | GO:0030054 | cell junction | 7.04 | 1.31.E-02 | 2.09.E+00 | 2.99.E-01 |
| CC | GO:0005925 | focal adhesion | 4.69 | 1.68.E-02 | 2.55.E+00 | 3.38.E-01 |
| CC | GO:0030426 | growth cone | 2.82 | 2.18.E-02 | 3.77.E+00 | 3.85.E-01 |
| CC | GO:0009986 | cell surface | 6.10 | 2.42.E-02 | 2.06.E+00 | 3.91.E-01 |
| CC | GO:0005794 | Golgi apparatus | 9.39 | 2.89.E-02 | 1.68.E+00 | 4.22.E-01 |
| CC | GO:0005886 | plasma membrane | 28.64 | 3.70.E-02 | 1.25.E+00 | 4.80.E-01 |
| CC | GO:0016021 | integral component of membrane | 38.50 | 3.73.E-02 | 1.19.E+00 | 4.60.E-01 |
| CC | GO:0005578 | proteinaceous extracellular matrix | 3.76 | 3.96.E-02 | 2.53.E+00 | 4.58.E-01 |
| CC | GO:0045121 | membrane raft | 3.29 | 4.78.E-02 | 2.67.E+00 | 5.04.E-01 |
| CC | GO:0035985 | senescence-associated heterochromatin focus | 0.94 | 4.89.E-02 | 3.99.E+01 | 4.92.E-01 |
| CC | GO:0031225 | anchored component of membrane | 2.35 | 5.28.E-02 | 3.54.E+00 | 5.00.E-01 |
| CC | GO:0046658 | anchored component of plasma membrane | 1.41 | 5.50.E-02 | 7.88.E+00 | 4.97.E-01 |
| CC | GO:0001726 | ruffle | 1.88 | 6.94.E-02 | 4.20.E+00 | 5.65.E-01 |
| CC | GO:0005783 | endoplasmic reticulum | 9.39 | 6.95.E-02 | 1.51.E+00 | 5.49.E-01 |
| CC | GO:0043025 | neuronal cell body | 4.69 | 8.74.E-02 | 1.87.E+00 | 6.19.E-01 |
| CC | GO:0005768 | endosome | 4.69 | 9.52.E-02 | 1.83.E+00 | 6.37.E-01 |
| MF | GO:0004553 | hydrolase activity, hydrolyzing O-glycosyl compounds | 2.35 | 5.77.E-04 | 1.29.E+01 | 2.09.E-01 |
| MF | GO:0005102 | receptor binding | 5.63 | 3.29.E-03 | 2.85.E+00 | 4.88.E-01 |
| MF | GO:0003680 | AT DNA binding | 1.41 | 3.52.E-03 | 3.27.E+01 | 3.79.E-01 |
| MF | GO:0005515 | protein binding | 27.23 | 4.77.E-03 | 1.39.E+00 | 3.84.E-01 |
| MF | GO:0016798 | hydrolase activity, acting on glycosyl bonds | 2.35 | 8.16.E-03 | 6.28.E+00 | 4.86.E-01 |
| MF | GO:0004860 | protein kinase inhibitor activity | 1.88 | 2.01.E-02 | 6.88.E+00 | 7.47.E-01 |
| MF | GO:0016491 | oxidoreductase activity | 6.10 | 2.04.E-02 | 2.11.E+00 | 6.98.E-01 |
| MF | GO:0005509 | calcium ion binding | 6.57 | 2.61.E-02 | 1.96.E+00 | 7.38.E-01 |
| MF | GO:0003705 | transcription factor activity, RNA polymerase II distal enhancer sequence-specific binding | 1.88 | 2.83.E-02 | 6.03.E+00 | 7.27.E-01 |
| MF | GO:0008449 | N-acetylglucosamine-6-sulfatase activity | 0.94 | 3.01.E-02 | 6.53.E+01 | 7.11.E-01 |
| MF | GO:0045499 | chemorepellent activity | 1.41 | 3.04.E-02 | 1.09.E+01 | 6.80.E-01 |
| MF | GO:0017080 | sodium channel regulator activity | 1.41 | 3.70.E-02 | 9.80.E+00 | 7.20.E-01 |
| MF | GO:0004566 | beta-glucuronidase activity | 0.94 | 4.00.E-02 | 4.90.E+01 | 7.20.E-01 |
| MF | GO:0008823 | cupric reductase activity | 0.94 | 4.00.E-02 | 4.90.E+01 | 7.20.E-01 |
| MF | GO:0000293 | ferric-chelate reductase activity | 0.94 | 4.00.E-02 | 4.90.E+01 | 7.20.E-01 |
| MF | GO:0052851 | ferric-chelate reductase (NADPH) activity | 0.94 | 4.00.E-02 | 4.90.E+01 | 7.20.E-01 |
| MF | GO:0001205 | transcriptional activator activity, RNA polymerase II distal enhancer sequence-specific binding | 1.41 | 4.65.E-02 | 8.65.E+00 | 7.48.E-01 |
| MF | GO:0086006 | voltage-gated sodium channel activity involved in cardiac muscle cell action potential | 0.94 | 4.97.E-02 | 3.92.E+01 | 7.48.E-01 |
| MF | GO:0004252 | serine-type endopeptidase activity | 2.82 | 6.50.E-02 | 2.77.E+00 | 8.18.E-01 |
| MF | GO:0019900 | kinase binding | 1.88 | 6.70.E-02 | 4.26.E+00 | 8.09.E-01 |
| MF | GO:0019838 | growth factor binding | 1.41 | 6.77.E-02 | 7.00.E+00 | 7.94.E-01 |
| MF | GO:0030374 | ligand-dependent nuclear receptor transcription coactivator activity | 1.41 | 7.05.E-02 | 6.84.E+00 | 7.90.E-01 |
| MF | GO:0019901 | protein kinase binding | 4.23 | 7.53.E-02 | 2.03.E+00 | 7.96.E-01 |
| MF | GO:0008046 | axon guidance receptor activity | 0.94 | 7.84.E-02 | 2.45.E+01 | 7.94.E-01 |
| MF | GO:0045545 | syndecan binding | 0.94 | 7.84.E-02 | 2.45.E+01 | 7.94.E-01 |
| MF | GO:0005178 | integrin binding | 1.88 | 8.15.E-02 | 3.92.E+00 | 7.92.E-01 |
| MF | GO:0004568 | chitinase activity | 0.94 | 9.70.E-02 | 1.96.E+01 | 8.35.E-01 |
| KEGG | mmu00531 | Glycosaminoglycan degradation | 2.35 | 8.63.E-05 | 2.03.E+01 | 1.46.E-02 |
| KEGG | mmu04142 | Lysosome | 3.29 | 2.80.E-03 | 4.90.E+00 | 2.13.E-01 |
| KEGG | mmu04360 | Axon guidance | 3.29 | 3.70.E-03 | 4.64.E+00 | 1.90.E-01 |
| KEGG | mmu05323 | Rheumatoid arthritis | 2.35 | 1.48.E-02 | 5.21.E+00 | 4.71.E-01 |
| KEGG | mmu00330 | Arginine and proline metabolism | 1.88 | 1.88.E-02 | 6.98.E+00 | 4.78.E-01 |
| KEGG | mmu05202 | Transcriptional misregulation in cancer | 2.82 | 4.20.E-02 | 3.11.E+00 | 7.06.E-01 |
| KEGG | mmu04145 | Phagosome | 2.82 | 4.77.E-02 | 3.00.E+00 | 6.97.E-01 |
| KEGG | mmu01100 | Metabolic pathways | 10.33 | 5.21.E-02 | 1.48.E+00 | 6.81.E-01 |
| KEGG | mmu05132 | Salmonella infection | 1.88 | 6.13.E-02 | 4.38.E+00 | 6.99.E-01 |
| KEGG | mmu04390 | Hippo signaling pathway | 2.35 | 9.73.E-02 | 2.83.E+00 | 8.26.E-01 |

BP: biological processes; CC: cellular components; MF: molecular function; KEGG: KEGG pathway.
